# Country-level effects of diagnosis-related groups: evidence from Germany’s comprehensive reform of hospital payments

**DOI:** 10.1007/s10198-023-01645-z

**Published:** 2023-12-05

**Authors:** Robert Messerle, Jonas Schreyögg

**Affiliations:** Hamburg Center for Health Economics, University of Hamburg, Esplanade 36, 20354 Hamburg, Germany

**Keywords:** DRG, Case-based payment, Hospital reimbursement, Hospital payment scheme, Hospital activity, Payment reform, H51, I11, I18, L51

## Abstract

Hospitals account for about 40% of all healthcare expenditure in high-income countries and play a central role in healthcare provision. The ways in which they are paid, therefore, has major implications for the care they provide. However, our knowledge about reforms that have been made to the various payment schemes and their country-level effects is surprisingly thin. This study examined the uniquely comprehensive introduction of diagnosis-related groups (DRGs) in Germany, where DRGs function as the sole pricing, billing, and budgeting system for hospitals and almost exclusively determine hospital revenue. The introduction of DRGs, therefore, completely overhauled the previous system based on per diem rates, offering a unique opportunity for analysis. Using aggregate data from the Organisation for Economic Co-operation and Development and recent advances in econometrics, we analyzed how hospital activity and efficiency changed in response to the reform. We found that DRGs in Germany significantly increased hospital activity by around 20%. In contrast to earlier studies, we found that DRGs have not necessarily shortened the average length of stay.

**Supplementary Information:**

The online version contains supplementary material available at 10.1007/s10198-023-01645-z.

## Introduction

Hospitals play a central role in healthcare provision, accounting for an average of 40% of total healthcare expenditure in Organisation for Economic Co-operation and Development (OECD) countries [1]. It is, therefore, not surprising that they are a prominent target for policy reform. In addition to the restructuring of hospitals and hospital care itself, the financing of hospitals is a recurring focus of policymakers. Indeed, changing the way in which hospitals are paid can influence the type and amount of care they provide, as well as the way in which they provide it. However, robust empirical evidence on the effects of different hospital payment schemes is scarce. Nevertheless, since the early 1980s, the vast majority of countries have adopted activity-based funding (ABF) as primary source of hospital financing, mainly in the form of case-based payments (CBP) using diagnosis-related groups (DRGs). DRGs group hospital cases into economically homogeneous groups based on their diagnoses. In doing so, DRG-based payment systems link hospital payments to the number of cases, with hospitals earning more by admitting and treating more patients. Moreover, when used as a pricing system, DRGs encourage hospitals to keep costs below the per-case flat rate, which in essence is a form of yardstick competition [2].

DRGs were first introduced in the United States (US) and gradually became the basis for hospital payment schemes around the world, albeit with country-specific adaptations. Depending on the previous payment system, the effects of such systems appear to work in opposite directions. The move from global budgets to DRG-based payment systems, as in most European countries, appears to have increased hospital activity and hospital expenditure. In contrast, in the US, where DRG-based hospital payments replaced fee-for-service payments, hospital activity initially decreased [3]. Although DRG-based payments are probably one of the most important health policy interventions in the past four decades, little is known about its system-wide effects at the country level.

In this paper, we use the introduction of DRGs in Germany (gDRGs) as a natural experiment to examine a particularly comprehensive reform of hospital payment. In 2004, Germany adopted DRG-based payments as the almost exclusive funding mechanism for all acute hospitals, going beyond partial implementations in other countries. In particular, to our knowledge, Germany stands out as the only country where DRGs-based payments are the sole basis for hospital pricing, billing, and budgeting, and account for 80% of total hospital reimbursement [4]. In many other countries DRG-based payments are mixed with other payment systems, so that DRG-based payments account for only a fraction of hospital revenues. As a result, Germany sets a remarkable benchmark for assessing the potential impact of a large-scale DRG reform and has also subsequently been a role model for other European countries.

To achieve a robust estimation of the effects of this reform, we used three complementary, quasi-experimental methods: difference-in-differences (DiD), synthetic control (SC), and synthetic difference-in-differences (SDiD). With aggregate country-level panel data for a comprehensive range of other countries and a classification of their hospital payment schemes, we were able to construct a suitable control group, allowing us to derive causal inference.

A major motive for introducing DRG-based payments was to increase hospital throughput by improving efficiency. Our main outcomes of interest were, therefore, related to hospital activity and efficiency, which we operationalized as hospital discharges and length of stay, respectively. Our approach allows us to complement the previous literature on DRG-based payments by providing effect estimates for a uniquely comprehensive reform. Our findings can also inform the ongoing policy discussion in Germany and elsewhere with robust evidence. We found that the introduction of gDRGs increased hospital discharges by more than 20% over ten years, approximately 2% annually. In contrast to previous studies, we were not able to identify any empirical evidence of an impact on the length of stay. Extensive robustness tests confirmed the validity of our results.

Our results add to the body of research on case-based payment systems, which separates into three basic streams (see Table 1). The first revolves around the effects of changes or reforms *within* case-based payment systems. Studies in this stream investigate hospitals’ responses to changes in prices or price structures within an existing case-based payment system (e.g., [5]). One of the main challenges here is to distinguish between effects at the intensive and extensive margins, for example between upcoding and genuine increases in the number of discharges. The majority of studies have found that hospitals react mainly by altering their coding practices, i.e., upcoding patients into higher-priced and therefore more profitable diagnoses [6–8]. Whether hospitals also alter treatment decisions in this setting remains unclear [9]. Some studies have found increases in the number of discharges for surgical but not medical DRGs [10], which is in line with some theoretical considerations [11]. Changes in the quality of care, for example in terms of in-hospital mortality, have not been found [12].

**Table 1:** Research streams on case-based payment systems

Research stream	Effects within case-based payment systems		Effects of case**-**based payment systems itself			
	Price changes		Hospital/specialty/patient level		System level	
Effect	Volume of care	+	Volume of care	0/+	Volume of care	0/+
	Quality of care	0	Length of stay	0/–	Length of stay	–
	Upcoding	++	Mortality	0	Mortality	0
			Quality of care	+/–	Quality of Care	0
			Readmissions or shift to post-acute care	++		
			Hospital efficiency	+/–		
Studies or reviews	[5, 7, 9, 24] and others		[13, 14] and others		[18–21]	
Caveats	Methodological challenges to differentiate between effects at the intensive and extensive margin		Short study periods, design constraints, e.g., lack of unaffected controls, availability of administrative data for pre-intervention period, econometric challenges		Payment scheme definition/specification of control group, sample size, econometric challenges	

– Decreasing, 0 no effects, + increasing, ++ strongly increasing. When several effects are listed, results are ambiguous

The second research stream investigates the effects of introducing a case-based payment system *itself*, focusing primarily on the level of *individual* hospitals, diseases or population subgroups. Overall, evidence in this stream suggests that introducing such a system causes substantial shifts to post-acute care and increases readmission rates. There is also evidence that the transition initially decreases the length of hospital stay [13]. However, the results of the studies in this stream are highly heterogeneous and limited by econometric challenges and design constraints [14], making it difficult to draw general conclusions. Indeed, non-experimental, descriptive studies—often covering only short periods—continue to predominate in this stream of the literature [15]. Even the studies that use more sophisticated econometric techniques are characterized by a high risk of bias [13]. Another design limitation is a lack of unaffected controls because most case-based payment systems have been implemented nationwide [16]. Lastly, appropriate data for pre-intervention periods are often lacking or of poor quality.

The third research stream, in which our study is situated, also analyzes the effects of introducing a case-based payment system, but at the *aggregate country level*. Research with this focus is necessary because even comprehensive studies from the previous research streams have generally been limited to subgroups of the population. Feess et al. found highly heterogeneous effects for certain subgroups following the introduction of the gDRG system [17]. At the aggregate level, however, they did not find any changes, which suggests that the heterogeneous results in the second research stream might be driven by the different scopes of analysis. To address this issue, studies in the third research stream have tried to establish a causal link between reforms of hospital payment schemes and subsequent developments using aggregate country-level panel data. To the best of our knowledge, only Moreno-Serra and Wagstaff [18], Wubulihasimu et al. [19] and, to some extent, Aragón et al. [20] and Farrar et al. [21] fall into this category. The first two estimated the effects of changes in hospital payment schemes, with case-based schemes as one example. Moreno-Serra and Wagstaff found that healthcare expenditure increased and length of stay decreased in their sample of Eastern European and Central Asian countries. Wubulihasimu et al. concentrated on OECD countries and found initial evidence of increased health expenditure and lower mortality; their results should be interpreted with caution, however, because they are sensitive to model specifications. Wubulihasimu et al. attributed the lack of unequivocal results to the heterogeneity of reforms and their only gradual or partial implementation. From a methodological point of view, both studies used, in part, the staggered difference-in-differences approach. Recent advances, however, suggest that this widely used approach can be biased when effect heterogeneity is present [22, 23] as it is the case for the many different payment reforms, opening room for further research.

Aragón et al. [20] and Farrar et al. [21] aggregated comprehensive micro-level data (with different time spans) to examine the impact of introducing DRG-based payments in England on length of hospital stay and other outcomes. They used similarly aggregated data from Scotland to construct a counterfactual. Both found profound decreases in the average length of stay. Farrar et al., moreover, found an increased volume of care [21].

Our paper is structured as follows. “Background” provides an overview of the gDRG system. “Data” follows with a description of the data and explains our approach to constructing a data set that covers the main hospital payment schemes in the control countries. “Methods” provides information about our methods and the estimation procedures. “Results” presents empirical results, and Sects. “Discussion” and “Conclusion” conclude.

## Background

Historically, hospitals in Germany have been restricted in their ability to provide outpatient care. Hospitals are therefore narrowly focused on inpatient care, which accounts for more than 90% of hospital revenues [25]. Outpatient care is instead mainly provided by independent physicians’ offices. However, the possibility for hospitals to perform selected outpatient procedures was introduced as early as 1992, but low outpatient reimbursement (today on average 25% of the inpatient revenue for the same procedure [26]) has largely prevented the international trend of increasing outpatient care and day care in hospitals in Germany.

For inpatient care, hospitals were for decades mainly paid by uniform per diem rates based on full cost compensation. In 1993, cost compensation wasabandoned and uniform per diem rates were then calculated on the basis of negotiated prospective budgets^1^. However, the prospective budgets were essentially still forward projected historical budgets. Lax budgeting rules moreover meant that hospital expenditure growth remained high.

Thus, the introduction of gDRGs in 2000 had three main objectives: to stabilize healthcare expenditures, to increase transparency concerning hospitals’ costs and activity, and to raise the efficiency by reducing the length of hospital stays. Despite the experience of other countries, the possibility that the system might lead to increased hospital activity was not a particular concern during the legislative process.

The gDRG system is based on the Australian Refined Diagnosis-Related Groups and was itself role model for the DRG system of Switzerland and Greece [27, 28]. It uses a grouping algorithm to assign cases to economically homogeneous DRGs based on criteria such as main diagnosis, medical procedures, and patient characteristics [29]. The base DRG is primarily determined by diagnoses and procedure codes; comorbidities and clinical characteristics are used to differentiate case severity. Initially, around 600 DRGs (including case severity splits) existed. This number almost doubled in a few years; more than thousand DRGs existed in 2008. The gDRG system is maintained and further developed by an independent institute under the supervision of the federal self-governing health care bodies^2^. In addition to maintaining the DRGs, the institute also calculates relative cost weights, which indicate the proportional cost of a gDRG compared to all other gDRGs. The calculations are based on retrospective cost and claims data collected by a sample of German hospitals [29]. The sample comprises approximately 15% of all hospitals, accounting for 20% of all cases. The data used to calculate costs are also the basis for the annual update of the DRG system, which involves medical, scientific and other external expertise in a structured dialog, for which any stakeholder can submit proposals.

To arrive at the final payment for a gDRG, the relative weight is multiplied by a base rate, which is negotiated—mainly along cost developments—at the federal state level by regional hospital associations and health insurers. The base rate is the same for all hospitals within a federal state and does not differentiate for rural/urban differences, the type of hospital, or any other difference between hospitals or regions. However, the base rate varies slightly from state to state, mainly due to historical rather than economic reasons.

Payments made under the gDRG system cover all operating costs. Additions or deductions are possible if the length of stay is above or below a DRG-specific threshold. The gDRG-based payment system is very comprehensive in scope: with the exception of some types of additional payment, for example for especially expensive medicines, it is the only pricing system used for hospitals in Germany [29]. DRGs are also used as basis for hospital budget negotiations and for direct billing purposes. Contingency costs, for example to ensure the provision of emergency care, are also included in DRG-based case payments. In contrast, the costs of long-term infrastructure investment are, in principle, financed by each of Germany’s 16 states through taxation. However, real public investment in hospitals has fallen steadily and now accounts for less than 5% of total hospital funding, a third of its 1991 level. DRGs allocate about 80% of all financial resources to hospitals. This is one of the highest shares among case-based hospital payment schemes internationally [4] and the reason why DRG-based payments are the main financial parameter for German hospitals.

The gDRG system became the mandatory inpatient payment system for all acute care hospitals in 2004. The introduction of the gDRG system consisted of two components. DRGs were combined with the introduction of state-wide prices to ensure equal prices at the regional level. However, to mitigate initial financial distortions, a gradual (financial) transition for hospitals took place. In the first year, hospital-specific base rates were calculated in such a way that the total payments received for a hospital’s case mix were the same as under the previous system. From 2005 onwards, the new system had financial consequences for hospitals, starting with base rates calculated as a mix of state-wide base rates (15%) and hospital-specific base rates (85%) [29]. From a system perspective, the base rates were neutral with some hospitals receiving higher and others lower base rates than the state average but the convergence until 2009 gave hospitals the opportunity to adjust to the state-wide uniform price system. Additionally, if a hospital suffered financial losses as a result of the reform (e.g., due to a lower number of cases than budgeted), the difference between budget and lower revenues was largely compensated. Losses to hospitals from the reform were therefore limited. On the other hand, gains from the reform, such as income from additional or more profitable cases, were largely uncapped. Thus, despite a transitional period, there were strong incentives for hospitals that benefited from the reform to increase their activity in the early years after the introduction of the new system. Effective regulation to reduce the volume incentives was introduced in 2017. In other words, from 2005 onwards, hospitals suddenly had the opportunity to generate substantial additional revenues through the gDRG system, which is why we consider its introduction as a binary treatment (see below).

Given this incentive structure, we would expect the effects to be broadly similar to those in other European countries. In line with yardstick competition, we would expect that a switch from per diem to case-based payments would lead to shorter lengths of stay to minimize costs and allow for additional cases. This effect should be reinforced by the relatively modest use of outpatient hospital care in Germany. As a result of the increased use of outpatient care in hospitals in other countries, the remaining inpatients would be expected to be more severe cases. In Germany, on the other hand, these patients remained as inpatients, reducing the average severity of inpatient cases. This should lead to a larger reduction in length of stay than in other countries. In terms of hospital volume, one would expect increased activity.

Although research has accompanied the gDRG reform throughout its implementation, clear causal evidence of such effects is lacking. As the gDRG system was mandatory and implemented nationwide, there is no suitable control group within the German health care system. As a result, most studies have resorted to describing trends only and have not been able to make statements about causality [30]. External time-varying factors, regression to the mean, false assumptions about the functional form of underlying time trends and other threats to internal validity render these single case time-series analyses problematic.

Based on their scoping review, Koné et al. concluded that although trends indicate that length of stay has decreased and case numbers have increased, there is no robust empirical evidence of either positive or negative effects of the introduction of DRGs in Germany [31]. Aggregate data also indicate that the average length of stay decreased, but less strongly than before the introduction of the gDRG system. In contrast, the number of cases and hospital expenditure increased. The number of inpatient discharges increased from 16.6 million in 2004 to 19.4 million in 2017, one of the largest increases in the number of hospital discharges in Europe, despite the fact that the population in Germany remained mostly stable (see online appendix A).

Given the lack of conclusive studies, policy discussions have taken place largely in the absence of evidence. In particular, the potentially negative consequences of DRG-based payment schemes on hospital care and staffing have attracted public attention. Ultimately, the assumption that financial incentives caused by the gDRG system had resulted in nurse understaffing, led to fundamental changes. With the passage of the so-called Nursing Staff Strengthening Act (Pflegepersonal-Stärkungsgesetz, PpSG), the German legislature decided in December 2018 that hospitals should be paid for the direct patient care provided by nursing staff independently of case payments. As a result, nursing costs, which accounted for around 20% of total DRG costs in 2017, are now excluded from DRG calculations. Thus, since 2020, hospital payments in Germany consist of a combination of per-case reimbursement via DRGs and a nursing staff allowance based on full cost compensation. From 2024 onwards, a further move away from the case-based payment system and toward a greater focus on prospective budgets is planned, with 60% of hospital revenues earmarked as fixed budget.

## Data

### Classification of hospital payment schemes

**Fig. 1:**
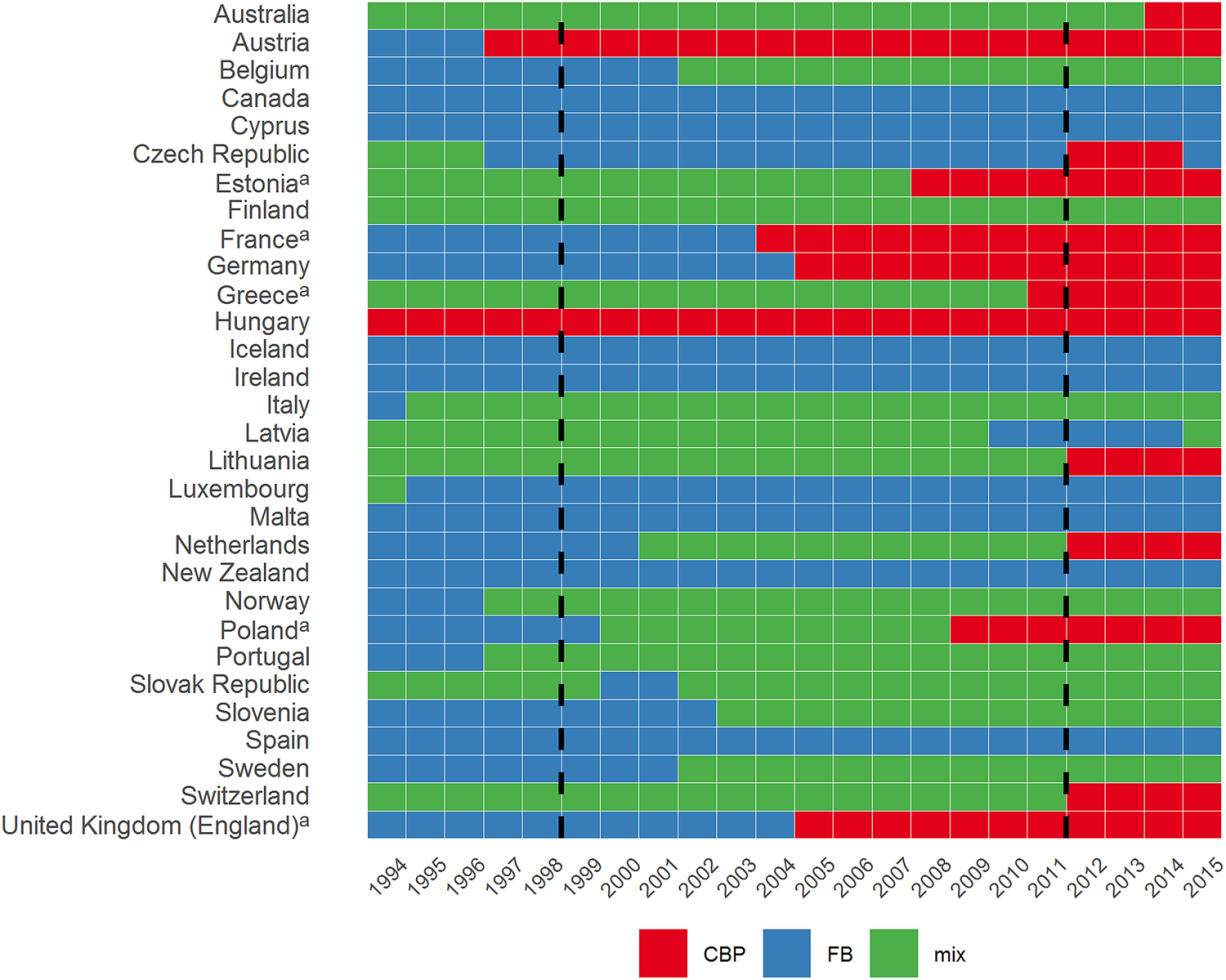
Hospital payment schemes in selected OECD and EU member states, 1994 to 2015. ^a^Countries that were not considered in the control and donor group because of a major reform with temporal proximity to the gDRG introduction. Notes: The black vertical lines illustrate a six-year time span around the gDRG introduction. See supporting information for further information regarding classification

Table 2 summarizes our data collection process and estimation strategy. To investigate the effects of introducing the gDRG system, we constructed—analogously to Moreno-Serra and Wagstaff [18] and Wubulihasimu et al. [19]—a control data set describing the main hospital payment schemes in selected OECD and EU member states from 1994 to 2015 (see Fig. 1). We included data from all European OECD countries and EU member states, as well as Australia, Canada and New Zealand. This sample therefore comprised high-income countries with generally comparable levels of healthcare provision and served as a starting point for constructing a suitable control group.

**Table 2:** Summary of statistical analysis

Step	Description
1. Collecting information on main hospital payment scheme in each country	We classified each country in our sample according to its main hospital payment scheme in each year from 1994 to 2015.
	First step: We assigned fixed budget (FB) or case-based payments (CBP) classification.
	(a) Our main source of information for classification was the Health Systems in Transition series. (b) Additional literature was used to supplement this information.
	Second step: We determined scope of payment scheme in order to distinguish between extensive and only partial reforms involving CBP.
	– CBP often only affects a fraction of hospital budgets (e.g., Denmark, Italy), is limited to certain hospitals and regions (e.g., Finland, Sweden) or is used for budgeting but not for actual billing processes (e.g., Ireland).
	We excluded all countries that introduced an extensive form of CBP between 1999 and 2011 from the control group. (Outside of this period, any reforms to hospital payment schemes should not bias the estimation)
2. Collecting and combining country-level data from several sources	We collected and combined (unbalanced panel) data from the OECD and Eurostat.
	(a) Main source for variables was the OECD (b) Data for additional countries from Eurostat
	Main outcome variables were: – Number of discharges per 100,000 inhabitants and – Length of hospital stay Secondary outcome variables concerned: – Hospital resources and expenditure as indicators of efficiency – Population health status Additional control variables, such as GDP per capita and share of population aged 65 or older, were used. Several variables exist in various definitions (see Supporting Information).
3. Applying three complementary estimation methods	We used different estimation methods to ensure our estimation was robust. (a) A slightly extended difference-in-differences (DiD) model was our baseline approach, which we used for all outcomes with a credible parallel trend assumption. (b) A synthetic control (SC) method was used for all outcome variables; the introduction of a DRG payment system is the prime example of a classic SC. (c) Synthetic difference-in-differences (SDiD) was used for all outcome variables.
4. Conducting robustness checks	We applied several robustness checks for the different methodological approaches. (a) Different control variables, control countries, parallel trend sensitivity analysis (b,c) Placebo-in-space and placebo-in-time analysis, different control countries To validate whether the introduction of DRGs in Germany was the driving force behind our effect estimates, we additionally checked for healthcare reforms that took place simultaneously. Because classifying hospital payment schemes was not straightforward for some countries, we also constructed an alternative classification scheme for the control countries.

First, to classify hospital payment schemes, we created two basic categories with opposite incentive structures: fixed budgets (FBs) and case-based payments (CBP), which differ from the classification used by Moreno-Serra and Wagstaff [18] and Wubulihasimu et al. [19]. We classified a country as using FBs in a given year if global budgets or block grants were the main form of hospital funding. In such cases, hospital revenue was determined mostly in advance based on provider characteristics like hospital size or the range of care provided. We classified a country as using CBP if hospitals were paid mainly according to the characteristics of the patients they admitted, for example payments based on a DRG classification. Our classifications were based on information from the Health System in Transition series of the European Observatory on Health Systems and Policies [32] and additional literature. More details can be found in the Supporting Information.

To help identify suitable control units, we added a mixed funding category to distinguish between extensive implementations of CBP and partial implementations co-existing with multiple payment schemes. Because many countries have implemented CBP schemes gradually or only partially, CBP often only affects a fraction of hospital budgets (e.g., in Denmark), is limited to certain hospitals and regions (e.g., in Finland and Sweden) or is used for budgeting but not for actual billing processes (e.g., in Ireland). We argue that in such cases the change in hospital incentive structures is considerably weaker, at least at the aggregate level used in our analysis. This approach allowed us to exclude countries that implemented reforms similar in scope to those in Germany from the control group while maintaining a reasonably large control group / donor pool.

For further analysis, we considered as control units all countries that did not introduce any major CBP reforms between 1999 and 2011, i.e., within a six-year period before and after the date of the gDRG reform. Reforms outside this period should not affect our estimates. As a result of this approach 24 countries remained as main control and donor group (see Fig. 1 for a complete list). However, we performed several robustness checks using control groups with different configurations.

To consider other kinds of major reforms that might affect hospital activity at the aggregate level, we screened the health policy literature for information on the relevant control countries and excluded these (i.e., Denmark) if necessary [33, 34]. We could not control, however, for smaller, gradual changes made to healthcare systems. Evidence on the effects of minor system changes has been inconsistent [35, 36] and it is implausible to expect pronounced effects at the aggregate national level in the absence of major reforms.

### Variables

We use unbalanced country-level panel data from OECD sources [37], complemented by data from Eurostat [38] and, for some economic indicators, from the World Bank [39]. Our main outcomes of interest were related to hospital activity and efficiency, which we operationalized as hospital discharges and average length of hospital stay. In line with previous research (see Table 1) and underlying incentive structures we expected decreases in length of stay and increased hospital activity. We also looked at secondary outcome variables which were related to hospital resources, healthcare expenditure and population health: the number of nurses and physicians employed by hospitals, inpatient expenditure, life expectancy, death rates and years of life lost. However, the results were inconclusive. We therefore report results only for our two main outcomes.

For our baseline model, we controlled for changes in GDP per capita to account for possible budgetary constraints caused, for example due to the impact of the financial crisis starting in 2007/2008. To capture time-varying effects on the demand side, we followed previous empirical work and used the share of the population aged 65 years or older [19]. Some of the additional variables used as controls could be endogenous, such as health care expenditure. In this case, lagged values were additionally considered as a robustness check. More details on the data can be found in the Supporting Information.

Table 3 gives an overview. Even at the aggregate level, certain health-related data were not available for all countries. Table 3 also underscores that Germany showed exceptionally high values for both outcomes before and after the introduction of the gDRG system. For our analysis, we have transformed all variables into natural logarithms for two reasons. First, we assume that the reform had a multiplicative effect depending on the base level. Second, to facilitate the interpretation of the results. However, the results for the outcomes as levels are included in the appendix and are generally comparable.

**Table 3:** Data description

	Variables	Germany		All other countries^a^		Control/donor group^a^	
		2004	2014	2004	2014	2004	2014
Outcomes	Hospital discharges	201	236	161 (28)	151 (29)	161 (23)	151 (24)
	Average length of stay	8.9	7.6	7.0 (28)	6.4 (29)	7.1 (24)	6.5 (24)
Others	Share of population 65 years or older	18 %	21 %	15 % (29)	17 % (29)	15 % (24)	17 % (24)
	GDP	37418	43561	34815 (29)	38001 (29)	36264 (24)	39613 (24)
	Hospital beds	6.4	6.2	4.5 (26)	3.7 (29)	4.6 (21)	3.6 (24)
	Private hospital beds	2.2	2.5	0.6 (12)	0.6 (18)	0.4 (9)	0.5 (15)
	Average idle bed capacity	1.5	1.3	1.1 (21)	1.0 (21)	1.1 (17)	0.9 (18)
	Healthcare expenditures	4156	5127	2960 (27)	3378 (29)	3105 (22)	3505 (24)
	Outpatient expenditures	978	1142	746 (24)	845 (28)	799 (20)	898 (23)

^a^In brackets: Number of countries with data available

Notes: Number of discharges and (idle) beds per 1000 inhabitants. Gross domestic product (GDP) and expenditures in US dollar per inhabitant

## Methods

### Empirical approach

The general objective of our approach was to obtain unbiased estimates of the effect of introducing the gDRG system. To achieve a robust estimation, we used three complementary methods:

- Difference-in-differences (DiD)
- Synthetic control (SC)
- Synthetic difference-in-differences (SDiD)

Although DiD and SC are normally used in different empirical settings, they are closely related [40]: a standard DiD approach can be considered an *unweighted* linear regression *with* unit and time fixed effects. Without covariates, it can be expressed as follows [40]:

$$\left( {\hat{\tau }^{{{\text{DID}}}} , \hat{\alpha }, \hat{\beta }} \right) = \mathop {\text{arg min}}\limits_{\alpha ,\beta ,\tau } \left\{ {\mathop \sum \limits_{i = 1}^{N} \mathop \sum \limits_{t = 1}^{T} \left( {Y_{it} - \alpha_{i} - \beta_{t} - W_{it} \tau } \right)^{2} } \right\},$$

with $Y_{it}$ being the outcome of interest, and $\alpha_{i}$ the unit fixed effects and $\beta_{t}$ the time fixed effects. $W_{it}$ denotes a binary intervention and $\tau$ the intervention effect. In contrast, the SC method [41, 42], which has been described as “arguably the most important innovation in the policy evaluation literature in the last 15 years” [43], drops the unit fixed effects $\alpha_{i}$ and instead adds unit weights $\hat{\omega }_{i}^{{{\text{SC}}}}$ to the regression function [40]. $\hat{\omega }_{i}^{{{\text{SC}}}}$ are restricted to be nonnegative and to sum to one. Weights are chosen so that the resulting weighted average best resembles the treated unit in terms of pre-treatment outcomes and covariates. SC can therefore be considered a *weighted* linear regression *without* unit fixed effects and can be expressed as follows:

$$\left( {\hat{\tau }^{{{\text{SC}}}} , \hat{\alpha }, \hat{\beta }} \right) = \mathop {\text{arg min}}\limits_{\alpha ,\beta ,\tau } \left\{ {\mathop \sum \limits_{i = 1}^{N} \mathop \sum \limits_{t = 1}^{T} \left( {Y_{it} - \beta_{t} - W_{it} \tau } \right)^{2} \hat{\omega }_{i}^{{{\text{SC}}}} } \right\}$$.

The third, very recently proposed method, SDiD, combines aspects of a standard DiD model and the SC estimator. Similar to DiD, it includes unit $\alpha_{i}$ and time $\beta_{t}$ fixed effects. Like SC, it uses unit weights $\hat{\omega }_{i}^{{{\text{SDID}}}}$ to align pre-intervention outcome trends among intervention and control units. In contrast to SC, however, SDiD allows for an intercept term in weight optimization. Thus, the pre-intervention outcomes of control and intervention units do not need to match exactly; instead, matching on trends is sufficient. SDiD additionally incorporates time weights $\hat{\lambda }_{t}^{{{\text{SDID}}}}$ to balance pre- and post-intervention periods. The time weights are chosen so that the weighted average of pre-intervention outcomes predicts the average post-intervention outcome for each control unit up to a constant. In this way, time weights can improve estimation by diminishing the influence of pre-intervention periods that are very different from post-intervention periods [40]. Both sets of weights are then used in a two-way fixed effects regression similar to DiD to obtain an estimate of the average causal effect of the intervention:

$$\left( {\hat{\tau }^{{{\text{SDID}}}} , \hat{\alpha }, \hat{\beta }} \right) = \mathop {\text{arg min}}\limits_{\alpha ,\beta ,\tau } \left\{ {\mathop \sum \limits_{i = 1}^{N} \mathop \sum \limits_{t = 1}^{T} \left( {Y_{it} - \alpha_{i} - \beta_{t} - W_{it} \tau } \right)^{2} \hat{\omega }_{i}^{{{\text{SDID}}}} \hat{\lambda }_{t}^{{{\text{SDID}}}} } \right\}$$.

Arkhangelsky et al. [40] demonstrated that SDiD has attractive properties with regard to bias and variance compared to the SC and DiD estimators.

In all three methods, high-income countries other than Germany that were not exposed to payment scheme reforms of similar extent function as a control group (see Fig. 1 and Supporting Information for a list of control units and additional information). The key assumption is therefore similar across all models: the outcome variables in Germany would have developed in ways similar to those seen in the (weighted set of) control countries if the gDRG system had not been introduced. Time of treatment is 2005, the first year with a financial impact for German hospitals.

#### Difference-in-differences

A DiD model represents our baseline. Despite an initial transition period, we have modeled the introduction of the gDRG system as a binary treatment. We believe this is appropriate because, despite this transition period, the new incentive structure for hospitals was in place immediately as mentioned above. Standard DiD models estimate one-time additive effects of a binary intervention at the outcome level. We deviated from a classic binary intervention and include interaction terms of treatment and time indicators ($Z_{it}$ = $W_{it} * \beta_{t}$) in our main model. In doing so, we followed previous research on the effects of payment scheme reforms, e. g. results by Aragón et al. [20] for England which highlighted the long-run effects of the English DRG reform starting in 2003. This approach is more similar to an event-study design and allows for lasting dynamic intervention effects.

With only one intervention unit and thus an absence of heterogeneous effects and varying timing, we did not have to consider recent insights on continuous interventions [44] or staggered DiD [22]. Our approach led to the estimation of the following equation:

$$Y_{it} = \alpha_{i} + \beta_{t} + \rho_{t} Z_{it} + \gamma X_{it} + \in_{it} ,$$

with $Y_{it}$ being the outcome of interest, $\alpha_{i}$ the country fixed effects, $\beta_{t}$ the time fixed effects, and $X_{it}$ the country-specific time-varying covariates. Countries and years are indexed by *i* and *t*. The identifying assumption is that potential outcomes without intervention evolve in parallel in the intervention and control groups after conditioning on observables (i.e., “parallel trends”).

#### Synthetic control method

Although it is currently applied to other settings and has undergone several methodological modifications (see [41] for an overview), SC was initially used to estimate the effects of aggregate interventions affecting only one individual unit [45, 46]. Analyzing the effects of introducing the gDRG system is, therefore, a prime example of its classic application. The underlying idea is that, at the aggregate level, a weighted combination of unaffected controls might provide better comparisons than individual unaffected units. The selection of controls is formalized by a data-driven procedure [41]. In contrast to a standard DiD model, SC does not depend on parallel pre-intervention trends in the outcomes of interest to justify its identification strategy. However, the identifying assumption is closely related. SC depends on finding a weighted set of control units that matches the intervention unit in the pre-intervention period as closely as possible (i.e., the “convex hull condition”). The identifying assumption then is that, in the absence of intervention, the intervention unit would have followed the weighted set’s outcome trajectory in the post-intervention period. Pre-intervention covariates were used in the weighting algorithm.

#### Synthetic difference-in-differences

We included the recently proposed *synthetic difference-in-differences* [40] framework which bridges both methods. SDiD compiles a weighted control group, assuring approximately parallel trends by virtue of its construction. Time weights diminish the role of periods that are very different from the considered post-intervention periods. Together, these adjustments make the estimation strategy more plausible and are similar to the current empirical practice of selecting suitable controls and periods a priori, but in a more transparent way. The identifying assumption is similar to that for SC: in the absence of intervention, the intervention unit would have followed the same outcome trajectory as that of the time- and unit-weighted set of control countries in the post-intervention period.

The principal setting for the SDiD framework includes only pre-intervention outcomes as predictor variables. Nonetheless, covariates $X_{it}$ can be incorporated by applying SDiD to the residuals of the regression of $Y_{it} on X_{it}$ [40].

Arkhangelsky et al. [40] presented different methods for inference, recommending a bootstrap or jackknife variance estimation to conduct asymptotically valid inference. However, both methods are designed for settings with large panels and many intervention units. For inference with $N_{tr} = 1$ (i.e., only one intervention unit) neither approach is well defined. As a third approach, Arkhangelsky et al. [40] constructed confidence intervals based on placebo evaluations, which are widely used in the SC framework. While this allows for some basic inference, this method probably leads to overly large confidence intervals.

### Comparison

All three methods have advantages and limitations. DiD is one of the most popular methods for causal estimation in health economics and beyond. Its data requirements are limited and its application and inference seem straightforward. In practice, however, not all requirements are usually met, and extensive recent research has shown that the consequences of violating basic assumptions have long been poorly understood. Most of these findings relate to heterogeneous treatment effects and variations in treatment timing [47]. While both are expected when looking at the effect at the level of individual hospitals, these methodological challenges do not apply in our case. As we use data at the national level and have only one treated unit, we have neither variation in the timing of treatment nor heterogeneous effects in our panel data. However, the sensitivity to non-parallel trends [48, 49] or to time-varying covariates [50] has also been of concern. A common way to assess the plausibility of the parallel trend assumption is to test for pre-intervention differences in trends. Recent papers have highlighted problems with this approach, e.g., its usually low power, and have underlined that more robust inference is needed [48, 51].

SC is one of the most prominent alternatives to DiD because of its easy interpretability and graphical explanation. However, it lacks an agreed statistical inference procedure and needs a good pre-treatment fit (“convex hull criterion”). Moreover, critics of SC argue that it provides specification-searching opportunities because its results can be highly volatile [52]. Discretionary choices made by the researcher with regard, for example, to the number of pre-intervention periods, the set of covariates or the exact specification of the weighting algorithm, can lead to widely different estimates because of the inherent sparsity of unit weights.

SDiD combines advantages from both approaches. Arkhangelsky et al. [40] demonstrated that their estimation approach is at least as good as DiD or SC with respect to variance and bias. However, statistical inference is so far not established and the properties of the estimator are not fully understood yet.

**Fig. 2:**
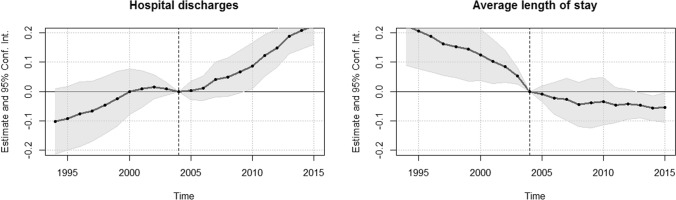
Event-study plot for the introduction of the German DRG system

In the case of our analysis, the assumption of parallel trends, judged by the widespread requirement of insignificant pre-treatment coefficients, holds for hospital discharges (see Fig. 2). However, as Fig. 2 also already indicates, recent research on sensitivity analysis following [51] confirms that the results are sensitive to the functional form of trends (see Online appendix C). The convex hull condition for SC is also difficult to guarantee for particular variables because the number of discharges and the average length of stay in Germany are both exceptionally high compared to other OECD countries (see Table 3), making it difficult to approximate Germany closely with a synthetic control. Considering all advantages and limitations, a combination of all three approaches is reasonable for our analysis.

All of our analyses were carried out using R, version 4.0.3. Fixest, Augsynth, and SynthDiD were primarily used as packages for estimation [40, 53, 54].

### Robustness checks

Although applying the different methods outlined above already provides a certain level of robustness, we nevertheless performed comprehensive additional checks. To begin with, we included additional control variables. On the supply side, we considered the number of hospital beds. Additionally, in Germany, it is often discussed that rising numbers of private hospital beds played a major role in increased hospital activity because private ownership reacts stronger to financial incentives. We therefore included these in the regression as well. Overall healthcare expenditure controls for varying scopes of healthcare cuts, for example due to the financial crisis beginning in 2007/2008. Furthermore, outpatient expenditure and the number of doctor consultations served as additional proxies for demand-side factors and possible shifts of activity to other sectors. We also tested whether our results critically depended on the choice of control countries. To this end, we used four different sets of control units. As described above, our main control group included all countries that did not introduce any extensive reform involving CBP between 1999 and 2011. Additionally, we built a set of control countries in which we excluded any country that we classified as having used CBP throughout our entire study period. A third, very narrow control group was based on FB classification only. For the fourth set of control countries, we used an alternative classification of payment schemes (see Supporting Information).

The gDRG system became the obligatory payment system for all acute care hospitals in 2004. The budgetary consequences started only in 2005, however, and were followed by a transition phase. Although we therefore used 2005 as the year of intervention in our main analysis, we repeated our analysis using different intervention timings as part of additional robustness checks (up to t=2009, see online appendix).

Within the SC framework, placebo tests are often recommended. The underlying idea is to replace the exposed unit with different units that were not exposed. By estimating a so-called placebo-in-space test, we were able to assess the magnitude of randomness in the data. Backdating the intervention is a similar approach, which can be applied to the SDiD framework as well. It works like a placebo-in-time test [41] and assigns a different intervention timing instead of a different intervention unit. If the estimation is able to reproduce the trajectory of the outcome variable prior to intervention and does not estimate any backdated effect, it adds credibility to the estimation itself [41]. In addition, we also used recent advances and provide results from a conformal inference method for SC [55].

Finally, to establish a plausible link between the changes in observed outcomes and the introduction of the gDRG system, we also discussed alternative explanations for our results.

## Results

### Difference-in-differences

The validity of DiD estimates is conditional on the assumption that the outcomes would have followed a common trend had the gDRG system never been introduced. Although this assumption itself is untestable, parallel pre-intervention trends can provide some reassurance. To this end, Fig. 2 includes an event-study plot with estimated coefficients and 95% confidence intervals for our outcomes.

The underlying regression equation is given by

$$Y_{it} = \alpha_{i} + \beta_{t} + \rho_{t} \left( {{\text{Treated}}_{i} \times {\text{Time}}_{t} } \right) + \gamma X_{it} + \in_{it} ,$$

where $\alpha_{i}$ and $\beta_{t}$ refer to country and time fixed effects, respectively. $\left( {{\text{Treated}}_{i} \, \times {\text{ Time}}_{t} } \right)$ refers to the interaction terms of intervention and time dummies. They replace the single intervention variable $Z_{it}$ and therefore lead to annual effect estimates $\rho_{t} .$ The pre-intervention coefficients $\rho_{{t\left[ {t < 2005} \right]}}$ for hospital discharges were not statistically significant, although for years before 2000 effect sizes were not near zero. For our second outcome, the average length of stay, there were clear signs of differing pre-trends. We, therefore, conclude that we can narrowly support the assumption of parallel trends for hospital discharges, but not for average length of stay.

The DiD regression results are shown in Table 4. Model 1 represents the baseline specification, controlling for changes in GDP per capita and the share of the population aged 65 or older. Models 2–5 add additional control variables. As described in the methods section, we included year-specific effect estimates to account for dynamic effects similar to the event-study-plot. A year-averaged treatment effect based on a 'classic' DiD, which is reported for completeness in Table 4, hides the dynamic effects of the policy and underestimates the impact.

**Table 4:** Difference-in-differences approach—estimated impact of introducing the gDRG system on hospital discharges

	Hospital discharges				
	(1)	(2)	(3)	(4)	(5)
Est. ρ for year = 2005	0.0514 (0.0446)	0.0419 (0.0490)	0.0022 (0.0248)	0.0902 (0.0618)	0.0518 (0.0324)
Est. ρ for year = 2006	0.0614 (0.0516)	0.0510 (0.0556)	0.0124 (0.0362)	0.1183 (0.0731)	0.0770 (0.0379)
Est. ρ for year = 2007	0.0935 (0.0593)	0.0734 (0.0645)	0.0438 (0.0457)	0.1532 (0.0845)	0.1233* (0.0473)
Est. ρ for year = 2008	0.1024 (0.0635)	0.0785 (0.0665)	0.0652 (0.0492)	0.1768 (0.0900)	0.1476** (0.0495)
Est. ρ for year = 2009	0.1216 (0.0658)	0.0879 (0.0707)	0.1230* (0.0529)	0.1893 (0.0935)	0.1796** (0.0518)
Est. ρ for year = 2010	0.1412 (0.0663)*	0.1022 (0.0708)	0.1359* (0.0607)	0.2058* (0.0975)	0.2025*** (0.0532)
Est. ρ for year = 2011	0.1759 (0.0597)**	0.1289 (0.0668)	0.1542* (0.0600)	0.2321* (0.0976)	0.2301*** (0.0549)
Est. ρ for year = 2012	0.1994** (0.0537)	0.1497* (0.0621)	0.1727** (0.0557)	0.2388* (0.0923)	0.2429*** (0.0546)
Est. ρ for year = 2013	0.2267 (0.0488) ***	0.1749** (0.0587)	0.1871** (0.0536)	0.2530** (0.0873)	0.2558*** (0.0510)
Est. ρ for year = 2014	0.2441*** (0.0471)	0.1903** (0.0567)	0.1925** (0.0493)	0.2587** (0.0823)	0.2648*** (0.0499)
Average treatment effect	0.1559** (0.0508)	*0.1192 (0.0583)*	0.1220* (0.0420)	0.1999* (0.0826)	0.1849*** (0.0458)
Observations	519	467	221	382	376
Controls for:					
Baseline	✓	✓	✓	✓	✓
Hospital beds		✓	✓	✓	✓
Private hospital beds			✓		
Healthcare and outpatient expenditure				✓	✓
Lagged values					✓

All estimates include country and year fixed effects, with outcomes and control variables in log form. Robust standard errors clustered at the country level in brackets. Results for average length of stay are not provided because we do not assume the parallel trend assumption to hold

***p < 0.001

**p < 0.01

*p < 0.05

The coefficients regarding hospital discharges remained stable in size and sign throughout all models. Thus, our estimation results seem robust to model alterations. The estimates for $\rho_{t = 2014}$, ten years after the gDRG introduction, ranged from 0.19 to 0.26 and were significant at the 1% level across all models. This implies an effect of around 20 % over ten years; an additional growth in the number of hospital discharges of 2% annually. We do not provide DiD results for our other main outcome, average length of stay, because the parallel trend assumption did not hold.

### Synthetic control

Table 6 summarizes the results of the SC approach. We again report effect sizes year by year as well as a year-averaged treatment effect. Inference was based on a conformal inference procedure [55]. We estimated synthetic controls individually for both outcomes and provide the corresponding weights in Online appendix B. Additionally, we employed a feature of the augmented synthetic control method [54] and estimated an SC model with both outcomes fitted in the weighting algorithm simultaneously (in which case inference is based on bootstraps). A RMSE placebo graph is provided in online appendix B and shows a reasonable fit of the base model.

The multiple outcomes approach resulted in a synthetic Germany composed of the Czech Republic (51%), Switzerland (22%), Austria (16%) and Norway (11%). Additional weights are provided in the Online appendix B. Due to Germany having one of the oldest populations worldwide and an abundant supply of hospital beds, a perfect fit was not possible (see Table 5).

**Table 5:** Comparison of real and synthetic Germany for *t*=2004

Country	Hospital discharges	Average length of stay	GDP	Share of population 65 years or older	Hospital beds	Average idle bed capacity
Germany	20,149.60	8.90	37,418.09	18.00	6.44	1.54
Synthetic Germany	19,967.03	9.36	34,260.57	14.65	5.35	0.94

*GDP* gross domestic product

Weights based on multivariate synthetic control model with hospital discharges and average length of stay as outcomes and GDP per capita and share of population aged 65 year or older as controls. Hospital beds shown only for comparison purposes. All variables re-transformed from natural logarithm

Table 6 shows the results. They are comparable in size and sign to those of our DiD estimates. The estimates for 2014, again ten years after the gDRG introduction, range from 0.12 to 0.27 and are significant in the multiple outcomes approach, but not individually for hospital discharges. The estimates for the average length of stay are close to zero and non-significant.

**Table 6:** Synthetic control approach—estimated impact of introducing the gDRG system

	Hospital discharges				Average length of stay			
	Single SC	Multi SC	Single SC with Controls	Multi SC with Controls	Single SC	Multi SC	Single SC with Controls	Multi SC with Controls
Est. for year = 2005	0.0059 (0.1705)	0.0023 (0.1040)	– 0.0062 (0.1671)	– 0.0841** (0.0299)	– 0.0214 (0.0203)	– 0.0396 (0.0984)	– 0.0087 (0.0138)	– 0.0110 (0.1090)
Est. for year = 2006	0.0103 (0.1705)	0.0167 (0.0997)	– 0.0036 (0.1671)	– 0.0695* (0.0318)	– 0.0049 (0.0203)	– 0.0341 (0.1043)	– 0.0073 (0.0138)	– 0.0062 (0.1124)
Est. for year = 2007	0.0400 (0.1705)	0.0413 (0.0872)	0.0242 (0.1671)	– 0.0360 (0.0443)	0.0209 (0.0203)	– 0.0176 (0.1034)	0.0013 (0.0138)	0.0014 (0.1047)
Est. for year = 2008	0.0662 (0.1705)	0.0705 (0.0728)	0.0481 (0.1671)	– 0.0042 (0.0491)	0.0126 (0.0203)	– 0.0238 (0.1061)	– 0.0037 (0.0138)	– 0.0004 (0.1087)
Est. for year = 2009	0.0941 (0.1705)	0.0711 (0.0439)	0.0753 (0.1671)	0.0139 (0.0476)	0.0205 (0.0203)	– 0.0232 (0.1244)	– 0.0031 (0.0138)	0.0033 (0.1248)
Est. for year = 2010	0.1234 (0.1705)	0.0942 (0.0349)**	0.1041 (0.1671)	0.0401 (0.0517)	– 0.0182 (0.0203)	– 0.0385 (0.1250)	– 0.0052 (0.0138)	0.0030 (0.1340)
Est. for year = 2011	0.1740 (0.1705)	0.1402*** (0.0245)	0.1553 (0.1671)	0.0865 (0.0625)	– 0.0336 (0.0203)	– 0.0534 (0.1332)	– 0.0166 (0.0138)	– 0.0051 (0.1534)
Est. for year = 2012	0.2063 (0.1705)	0.1565*** (0.0330)	0.1923 (0.1671)	0.0999 (0.0543)	– 0.0193 (0.0203)	– 0.0392 (0.1316)	– 0.0035 (0.0138)	0.0117 (0.1558)
Est. for year = 2013	0.2346 (0.1705)	0.1668*** (0.0336)	0.2376 (0.1671)	0.1134* (0.0474)	– 0.0202 (0.0203)	– 0.0420 (0.1297)	– 0.0076 (0.0138)	0.0089 (0.1585)
Est. for year = 2014	0.2617 (0.1705)	0.1764 (0.0382) ***	0.2710 (0.1671)	0.1243** (0.0413)	– 0.0232 (0.0203)	– 0.0502 (0.1378)	– 0.0173 (0.0138)	0.0008 (0.1673)
Average treatment effect	0.1364 (0.0506) *	0.1035* (0.0514)	0.1263** (0.0476)	*0.0428 (0.0426)*	*– 0.0088 (0.1183)*	*– 0.0377 (0.1196)*	*– 0.0097 (0.7706)*	*– 0.0019 (0.1300)*
Variables used in weight determination:								
Baseline	✓	✓	✓	✓	✓	✓	✓	✓
Hospital Beds, Healthcare Expenditure and Outpatient Expenditure			✓	✓			✓	✓

*SC* synthetic control, *Single SC* Outcome-specific control group weights, *Multi SC* One set of control group weights across multiple outcomes

All estimates with outcomes and control variables in log form. Approximated standard errors based on the conformal inference procedure from Chernozhukov et al. (2021) are shown in brackets

***p < 0.001

**p < 0.01

*p < 0.05

### Synthetic difference-in-differences

Table 7 shows the results for the SDiD approach. We restricted our estimations to the baseline control variables (GDP and share of the population aged 65 years or older). Because SDiD needs balanced panel data, the inclusion of additional control variables with limited availability would have decreased the size of the donor pool for control units prohibitively. The SDiD approach distributes weights across many countries. For hospital discharges, no country was weighted more than 20%, with Austria, Spain, Norway, and Hungary receiving the highest weights. For average length of stay, no country was weighted more than 9%. Time weights, on the other hand, were very sparse. We provide the weights in online Annex B.

**Table 7:** Synthetic difference-in-differences approach—estimated impact of introducing the gDRG system

	Hospital discharges	Average length of stay
Est. for year = 2005	– 0.0087 (0.1061)	0.0028 (0.1031)
Est. for year = 2006	0.0080 (0.1061)	0.0031 (0.1031)
Est. for year = 2007	0.0570 (0.1061)	0.0077 (0.1031)
Est. for year = 2008	0.0767 (0.1061)	– 0.0093 (0.1031)
Est. for year = 2009	0.1008 (0.1061)	– 0.0004 (0.1031)
Est. for year = 2010	0.1207 (0.1061)	– 0.0028 (0.1031)
Est. for year = 2011	0.1597 (0.1061)	– 0.0487 (0.1031)
Est. for year = 2012	0.1909* (0.1061)	– 0.0575 (0.1031)
Est. for year = 2013	0.2068* (0.1061)	– 0.0691 (0.1031)
Est. for year = 2014	0.2181* (0.1061)	– 0.0916 (0.1031)
Average treatment effect	*0.1248 (0.1013)*	*– 0.0305 (0.0914)*

*gDRG* German (system of) diagnosis-related groups

All estimates with outcomes and control variables in log form. GDP per capita and share of population aged 65 years or older used as control variables. Standard errors from placebo evaluations in brackets

***p < 0.001

**p < 0.01

*p < 0.05

The estimates were similar in sign and size to our previous results. As described in the methods section, the confidence intervals were based on placebo evaluations [40]. This method generally leads to rather large intervals. Notwithstanding these limitations, the results of the SDiD approach broadly confirmed our findings. It produced a significant point estimate for hospital discharges of 0.22 for 2014. For the average length of stay the results were not significant but from 2011 on showed negative (still non-significant) effects.

### Further robustness checks

The steps described in the sections above already included initial robustness checks. To ensure comprehensiveness we ran several additional checks, focusing on our main outcome, hospital discharges, because other estimates were inconclusive. The results of our robustness checks underline the validity of our results (see online appendix C).

### Summary

**Fig. 3:**
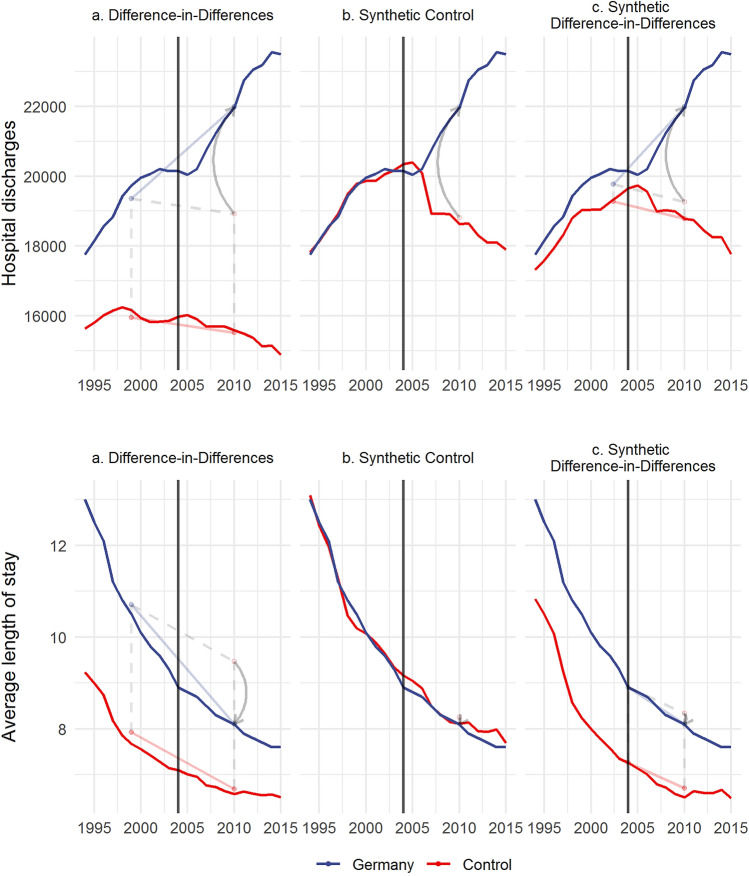
Illustrated impact of the gDRG introduction for all three methods. The blue line shows the trajectory of our two outcomes for Germany and is similar for all three methods (without covariates due to methodological constraints). The red line represents the trajectory of counterfactual Germany based on the control group and differs according to each of the three methods. Hospital discharges per 1000 inhabitants, average length of stay in days. The parallelogram shows the change from the weighted pre-treatment average to the post-treatment average for Germany and the control group. The arrow represents the resulting (average) treatment effect. The vertical line indicates last pre-treatment year. Weights are provided in online appendix

For illustrative purposes, Fig. 3 plots the results for our two main outcomes for all three methods.^3^ Overall, the results are comparable. All methods found unequivocal effects for hospital discharges. The results indicate that introducing the gDRG system increased the number of hospital discharges by more than 20% over ten years, or approximately 2% per year. For the average length of stay, we were not able to identify a consistent effect using SC or SDiD, and the DiD results were not credible due to differential pre-trends.

A closer look at the weights emphasizes the differences among our estimation approaches (see online appendix B). Because the optimization procedure of SC assigns zero weights to several control units, the control group for one outcome can be very different from the control group for another outcome. Slight changes, such as which covariates are included, which period is considered, or which weight optimization algorithm is used, can lead to very different control unit weights. Thus, the results are highly responsive to small variations. In contrast, SDiD and DiD use comparatively distributed or uniform weights and are therefore less affected by individual country comparisons.

## Discussion

With this study, we contribute to the scarce body of literature on the aggregate country-level effects of hospital payment schemes by investigating the impact of introducing DRGs in Germany. To date, the most commonly used methods for assessing the effects of such payment schemes have been non-experimental and descriptive, and recent reviews have highlighted the need for quasi-experimental approaches [15]. Our study helps address this gap by applying recent econometric advances to analyze the effects of a uniquely comprehensive reform involving the introduction of DRGs.

We used aggregate country-level data to examine changes in German hospital activity despite not having a control group in Germany itself. Due to the panel structure of our data, we were able to control for underlying aggregate trends in economic and demographic variables, as well as unobserved across-country influences. The case of Germany is particularly interesting for two reasons. First, in the last two decades, it has experienced one of the largest rises in the number of hospital discharges among OECD and EU countries which has not yet been not fully explained. Second, many countries have only partially implemented DRGs, either for certain aspects of hospital financing or limited to certain regions. In contrast, the German DRG system is one of the most comprehensive implementations of case-based financing in the world, as few other countries have introduced DRGs on such a large scale, with 80% of total hospital reimbursement being allocated through DRGs, and with DRGs also being used for pricing rather than primarily for budgeting. The German DRG system functions as the sole pricing, billing and budgeting system throughout the country and almost exclusively determines hospital revenues. This makes Germany a remarkable benchmark for assessing the potential impact of a large-scale DRG reform. We believe that our results provide an upper bound on the potential impact of DRG reform which has also served as a role model for several other countries, such as Greece and Switzerland.

Our triple quasi-experimental estimation approach allowed us to combine the individual advantages of each method and generate an ensemble estimate. While each of the methods perform differently depending on the real data generating process, together their results indicate that introducing the gDRG system led to a sharp increase in the number of hospital discharges. Indeed, we estimated a prolonged effect of approximately 20% over our estimation period of 10 years. That means that the number of discharges increased by more than 2% annually. To put this into perspective: based on the average case costs in 2005 and all else being equal, this amounts to hospital expenditures of over one billion euros. Starting from an already very high level of hospital activity, introducing the gDRG system put an even stronger focus on inpatient care. This finding is somewhat contrary to previous studies of the introduction of case-based systems, which have not identified unequivocal evidence of an increase in the volume of care [13, 14]. However, the majority of these studies analyzed specific procedures and diagnoses rather than the aggregate impact of reforms [13]. Importantly, our results are robust across all models and methods.

In contrast to some of the previous literature, we were not able to identify any effects on the length of hospital stay. Although the absence of evidence is not evidence of absence, one would in theory expect to find pronounced declines given the incentive structure of the gDRG system. Internationally, a majority of studies have found a connection between DRGs and declines in length of stay [20, 31, 56]. However, the few studies for Germany have often not considered existing pre-trends (for example [57, 58]). For the closely related introduction of DRGs in Switzerland, an impact on the length of stay was not found [27]. Moreover, a more recent study on the introduction of the gDRG system using quasi-experimental approaches also did not find any aggregate effect on the length of stay [17]. Instead, the length of stay changed only for subsets of the population depending on patient and hospital characteristics.

The reason for our unexpected result remains open. One reason might be the high idle hospital capacity in Germany (see Table 3). In contrast to other countries, hospital waiting times, e.g., for surgery, do not pose a problem. Due to abundant technical resources, German hospitals did not have to reduce the length of stay in response to the economically induced increase in the number of discharges. Our results would seem to suggest that the overall prevailing trend of shorter stays was sufficient to accommodate more patients.

Our study has a number of important limitations that must be considered when interpreting our results. First, the analysis is conducted at the macro level, which means that there is only one country as treatment group and possible heterogeneous lower-level and subgroup effects could not be analyzed. To overcome this limitation and analyze data at the micro level, not only hospital-level data from Germany but also from other countries for a period of 20 years would be needed to construct a suitable control group. In the absence of such sufficient micro data, several papers have used aggregated country-level OECD data to analyze the effects at the national level [19, 59, 60].

Second, even at the aggregate level, only limited data were available for the period considered. Depending on the variable, the earliest data were from the mid-1990s. For many variables, however, there were little pre-intervention data, and this was sometimes limited to selected countries. Data scarcity, therefore, influenced our choice of controls. Estimating counterfactuals based on a more comprehensive set of data might have led to different estimates. Nevertheless, the use of country-level data limits the extent to which low-level data errors can affect the estimation. Data quality issues may affect analyses at the level of individual hospitals or cases, but are averaged out at the country level.

Third, other unobserved factors may have influenced hospital activity in Germany and the control countries. We included several variables to control for time-varying factors. However, there are limits to the extent to which control is possible with aggregated data. Finally, the construction of appropriate control groups is a limitation in itself. The classification of payment schemes we used to define appropriate controls was not always straightforward, and any assignment will always be somewhat arbitrary.

## Conclusion

Research on the effects of hospital payment reforms is surprisingly scarce. Our paper helps to fill this gap in the literature by using a triple quasi-experimental estimation approach to analyze an especially comprehensive DRG-based payment scheme introduced in Germany. To the best of our knowledge, we provide the first cross-country empirical analysis of this reform in Germany. Using aggregate panel data, we found a pronounced effect on hospital activity in the form of a large increase in the number of hospital discharges. Somewhat unexpectedly, we did not find any evidence of a decrease in the length of hospital stay.

Our results complement two different strands of the literature. First, they add to the ongoing policy discussion on the long-term effects of the gDRG system. For German hospitals, DRGs are the almost exclusive source of revenue. This is why the change in financial incentives entailed in the reform induced a steep increase in the number of hospital discharges. However, German hospitals are also equipped with high (idle) capacities, which presumably explains why the reform did not reduce the overall length of stay. In short, based on our results and judged only at the aggregate level, the introduction of the gDRG system in Germany failed to achieve one of its major goals.

Second, our results also add to the overarching literature on the impact of case-based payments by assessing the effects of a large-scale DRG reform. Our evidence suggests that DRGs can lead to an increase in discharge rates but do not necessarily decrease the length of stay. By using quasi-experimental approaches, our research adds relevant insights to the literature on the aggregate country-level effects of hospital financing. However, given the limitations of our study, these findings must still be interpreted with caution.

Overall, our results suggest that hospitals do indeed respond to incentives induced by payment reforms and that the effects are visible even at the aggregate level. However, the direction and magnitude of the response are sensitive to the health system’s context. Policy makers should be cautious when assuming that the effects of interventions in one jurisdiction can be replicated easily in others. The introduction of DRGs in Germany underscores the possibility that complex interventions can have unexpected consequences in a different context.

### Supplementary Information

Below is the link to the electronic supplementary material.

Supplementary file1 (PDF 1816 KB)

Supplementary file2 (PDF 678 KB)

## Data Availability

The data that support the findings of this study are openly available from OECD [37], Eurostat [38] and World Bank [39].

## Footnotes

1 A small proportion of hospital revenues (less than 20%) was provided by case-based payments.

2 The National Association of Statutory Health Insurance Funds, the Association of Private Health Insurance, and the German Hospital Federation.

3 Without covariates because within the SDID framework [40] these are incorporated by applying SDiD to the residuals of the regression of Y_it_ on X_i_. Outcome trajectories are therefore not directly comparable in models with time-varying covariates. The corresponding weights are provided in the Online appendix.
