# Supplementary material for: Country-level effects of diagnosis-related groups: evidence from Germany’s comprehensive reform of hospital payments
Source: Eur J Health Econ. 2023 Dec 5;25(6):1013–30. doi: 10.1007/s10198-023-01645-z (PMC11283398; doi:10.1007/s10198-023-01645-z)
Supplement: Supplementary file 1 — Supplementary file1 (PDF 1816 KB) [file 10198_2023_1645_MOESM1_ESM.pdf]

Online-Appendix

Country-level effects of diagnosis-related groups: Evidence from Germany's comprehensive reform of hospital payments

Robert Messerle and Jonas Schreyögg[1]

Hamburg Center for Health Economics, University of Hamburg, Esplanade 36, 20354, Hamburg, Germany

In: European Journal of Health Economics

[1]Correspondence to Jonas Schreyögg, University of Hamburg, Esplanade 36, 20354, Hamburg, Germany.
Email: jonas.schreyoegg@uni-hamburg.de, Phone: +49 42838-8041

Appendix A – General

Trajectory in Germany compared to the other European countries

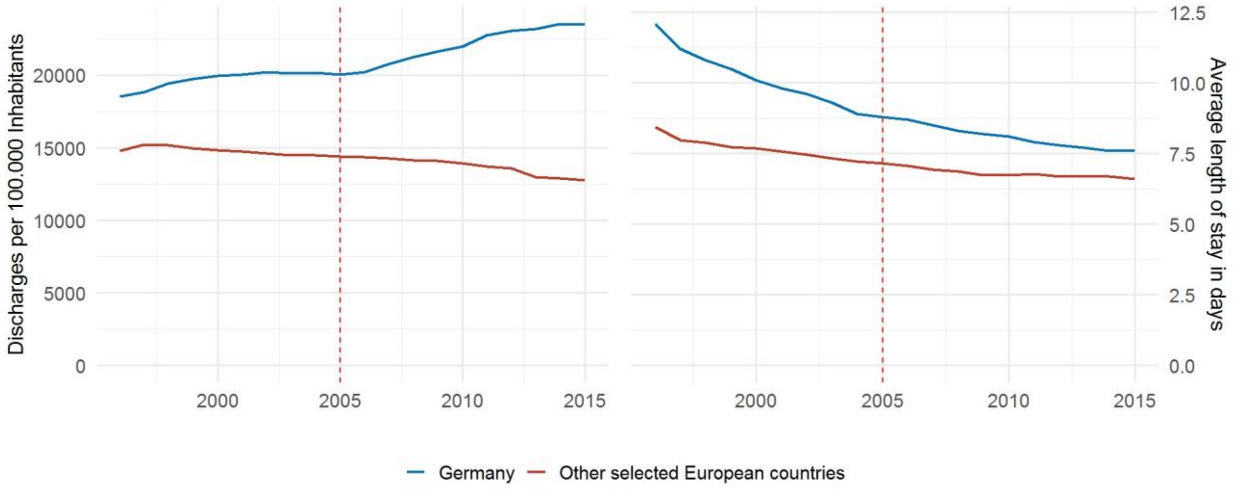

FIGURE A1. Curative care hospitalizations and length of stay.

Source: OECD 2021

Notes: Other selected European countries are all European countries from the main control group.

Non-hospital ambulatory care exposure

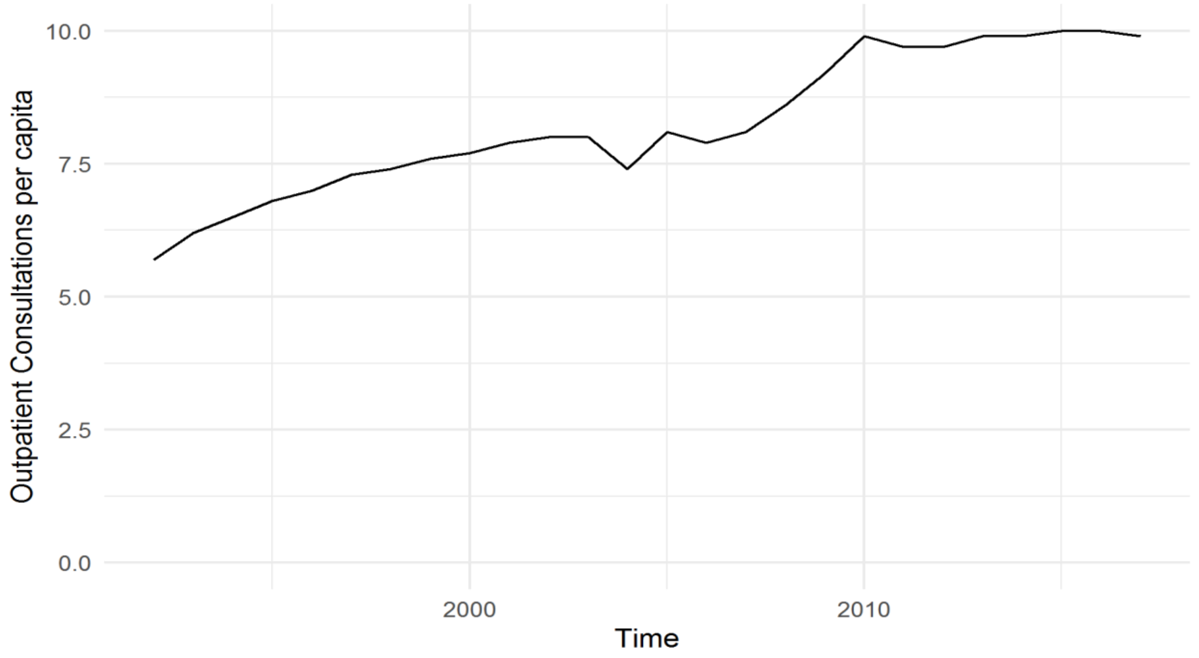

FIGURE A2. Annual number of ambulatory doctor consultations per capita in Germany

Source: OECD 2021

Appendix B – Additional results

SC country weights

Country	Hospital Discharges	Average Length of Stay	Multiple (main) Outcomes
AUS			
AUT	0.59		0.16
BEL			
CAN			
CHE		0.69	0.22
CYP			
CZE		0.26	0.51
ESP	0.14		
FIN			
HUN			
IRL			
ISL			
ITA	0.19		
LTU		0.05	
LUX			
LVA			
MLT			
NLD			
NOR	0.03		0.11
NLZ			
PRT			
SVK			
SVN			
SWE	0.05		

TABLE B1. Synthetic control approach - Weights for baseline model

SDiD country weights

Country	Hospital discharges	Average length of stay
AUS	0.00	0.03
AUT	0.17	0.05
BEL		
CAN	0.00	0.01
CHE	0.00	0.05
CYP	0.06	0.06
CZE	0.04	0.05
ESP	0.15	0.05
FIN	0.03	0.00
HUN	0.13	0.07
IRL	0.00	0.02
ISL	0.00	0.09
ITA	0.02	0.05
LTU	0.15	0.08
LUX		
LVA		
MLT		0.00
NLD	0.00	0.09
NOR	0.13	0.05
NZL		
PRT	0.04	0.04
SVK	0.00	0.08
SVN	0.08	0.09
SWE	0.00	0.04

TABLE B2. Synthetic difference-in-differences approach – Country weights for baseline model

SDiD time weights

Year	Hospital Discharges	Average Length of Stay
1994	0.08	0.00
1995	0.00	0.00
1996	0.00	0.00
1997	0.00	0.00
1998	0.00	0.00
1999	0.00	0.00
2000	0.00	0.00
2001	0.00	0.00
2002	0.00	0.00
2003	0.00	0.00
2004	0.92	1.00

TABLE B3. Synthetic difference-in-differences approach – Time weights for baseline model

Corresponding weights for figure 3

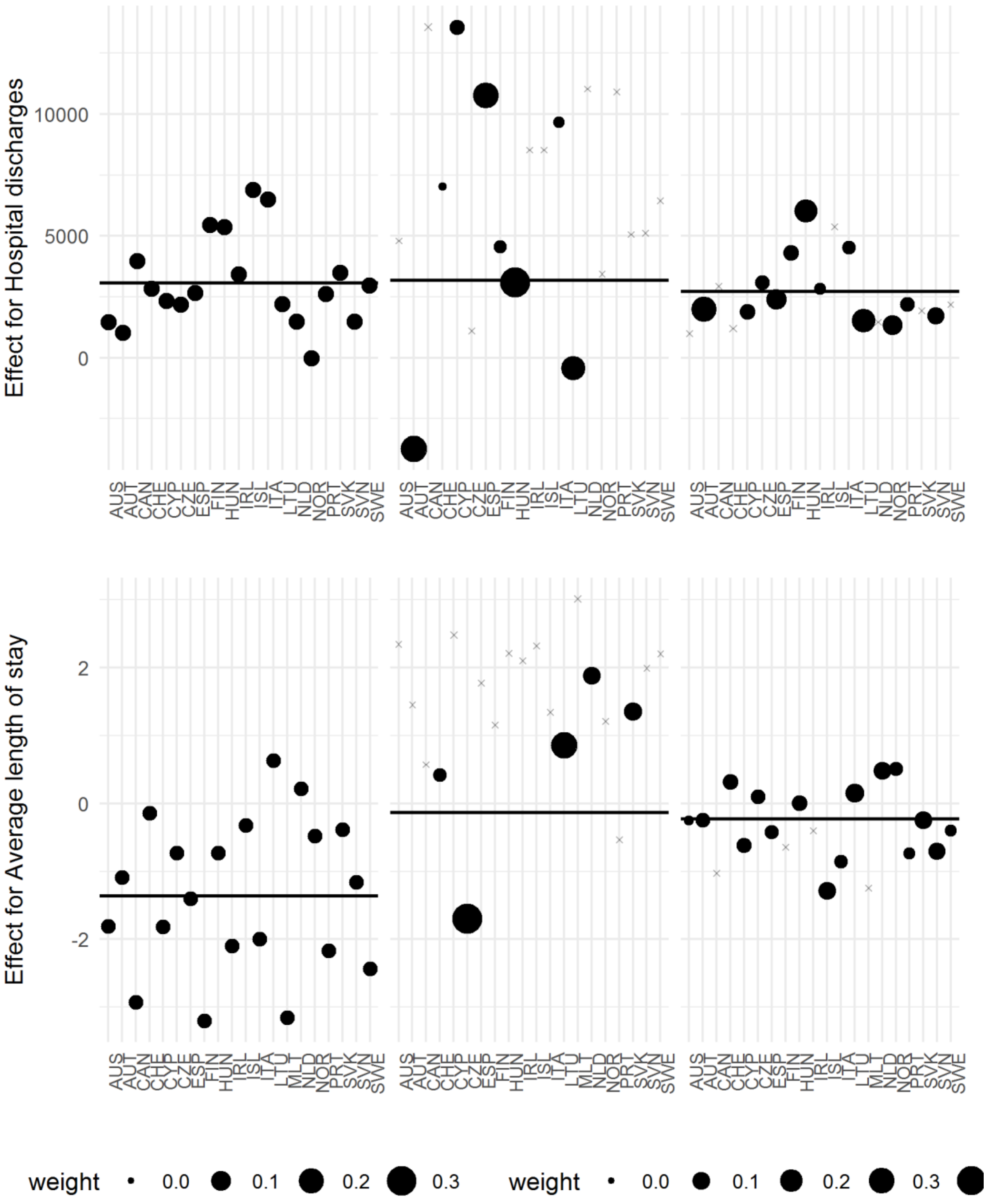

FIGURE B1. Corresponding weights for figure 3

DiD results for outcomes as levels

	(1)	(2)	(3)	(4)	(5)
Est. ρ for year = 2005	911.5 (632.6)	1,036.7 (785.9)	-85.44 (530.3)	1,470.6 (995.7)	842.0 (576.1)
Est. ρ for year = 2006	1,099.7 (742.1)	1,262.1 (889.8)	296.5 (724.2)	1,950.6 (1,190.0)	1,253.3 (694.2)
Est. ρ for year = 2007	1,771.1 (885.5)	1,868.4 (1,053.7)	1,034.3 (1,022.4)	2,677.6 (1,405.2)	2,157.2* (873.3)
Est. ρ for year = 2008	2,087.0* (956.5)	2,158.3 (1,096.3)	1,486.6 (1,110.5)	3,138.6* (1,516.3)	2,691.6** (915.6)
Est. ρ for year = 2009	2,550.2* (1,042.9)	2,562.8* (1,192.4)	2,482.3 (1,255.3)	3,448.2* (1,600.8)	3,304.7** (952.7)
Est. ρ for year = 2010	2,922.2* (1,107.9)	2,856.0* (1,214.2)	2,799.3* (1,261.4)	3,764.6* (1,708.6)	3,809.3*** (984.1)
Est. ρ for year = 2011	3,573.7** (1,028.8)	3,347.2** (1,147.0)	3,301.6* (1,198.4)	4,288.8* (1,730.0)	4,482.1*** (1,000.2)
Est. ρ for year = 2012	3,974.4*** (934.2)	3,697.0** (1,050.1)	3,506.2** (1,062.2)	4,490.2* (1,629.8)	4,697.6*** (973.3)
Est. ρ for year = 2013	4,397.8*** (858.0)	4,098.2*** (979.1)	3,815.1** (975.9)	4,725.4** (1,535.3)	4,935.1*** (921.0)
Est. ρ for year = 2014	4,775.4*** (798.4)	4,455.9*** (906.4)	4,111.3*** (921.9)	4,894.8** (1,424.0)	5,205.9*** (893.3)
Average Treatment Effect	*3,035.5** (815.6)*	*2,926.3** (958.9)*	*2,358.1* (817.5)*	*3,578.3* (1,408.4)*	*3,475.7*** (830.4)*
Observations	519	467	239	382	376
Controls for:					
Baseline	✓	✓	✓	✓	✓
Hospital Beds		✓	✓	✓	✓
Private Hospital Beds			✓		
Healthcare and Outpatient Expenditure				✓	✓
Lagged Values					✓

*** p < 0.001, ** p < 0.01, * p < 0.05
Notes: All estimates include country and year fixed effects, with outcomes and control variables as levels. Robust standard errors clustered at the country level in brackets. Results for average length of stay are not provided because we do not assume the parallel trend assumption to hold.

TABLE B4. Difference-in-differences approach – results for outcomes as levels

SC results for outcomes as levels

	Hospital discharges				Average length of stay			
	**Single SC**	**Multi SC**	**Single SC with Controls**	**Multi SC with Controls**	**Single SC**	**Multi SC**	**Single SC with Controls**	**Multi SC with Controls**
Est. ρ for year = 2005	-362.4239 (3083.2344)	-1794.4622 (1015.0888)	-48.2979 (3289.5918)	-235.2317 (1426.2539)	-0.1428 (0.2814)	-0.1836 (0.8597)	-0.2576 (0.2113)	-0.2778 (0.8409)
Est. ρ for year = 2006	-358.4885 (3083.2344)	-1496.0584 (1060.9966)	9.9013 (3289.5918)	178.6341 (1353.4788)	-0.1728 (0.2814)	-0.1403 (0.8788)	-0.1461 (0.2113)	-0.2100 (0.8710)
Est. ρ for year = 2007	156.6918 (3083.2344)	-868.3423 (1129.8050)	572.1367 (3289.5918)	703.9555 (1139.3864)	-0.0585 (0.2814)	-0.0375 (0.7970)	0.0651 (0.2113)	-0.0656 (0.8100)
Est. ρ for year = 2008	609.1808 (3083.2344)	-215.0697 (1057.9777)	1083.9296 (3289.5918)	1264.3300 (916.7015)	-0.1349 (0.2814)	-0.0577 (0.8131)	0.0020 (0.2113)	-0.0676 (0.8241)
Est. ρ for year = 2009	1149.2778 (3083.2344)	212.3847 (891.0860)	1661.9124 (3289.5918)	1367.7315* (532.8785)	-0.1776 (0.2814)	-0.0459 (0.9347)	0.0497 (0.2113)	-0.0087 (0.9541)
Est. ρ for year = 2010	1722.0054 (3083.2344)	779.0007 (860.7203)	2266.8081 (3289.5918)	1818.0862*** (441.1916)	-0.2257 (0.2814)	-0.0717 (0.9893)	-0.2100 (0.2113)	-0.0874 (0.9941)
Est. ρ for year = 2011	2747.3747 (3083.2344)	1798.5013 (946.4423)	3322.3692 (3289.5918)	2617.2604*** (606.9652)	-0.3684 (0.2814)	-0.1986 (1.1099)	-0.3446 (0.2113)	-0.1851 (1.0540)
Est. ρ for year = 2012	3527.8801 (3083.2344)	2153.1637** (833.9507)	4003.4997 (3289.5918)	2981.2346*** (663.1078)	-0.2912 (0.2814)	-0.0734 (1.0910)	-0.2291 (0.2113)	-0.0535 (1.0148)
Est. ρ for year = 2013	4414.6318 (3083.2344)	2520.2134** (864.5820)	4550.6733 (3289.5918)	3245.5190*** (626.0801)	-0.3322 (0.2814)	-0.1082 (1.0987)	-0.2419 (0.2113)	-0.0783 (0.9882)
Est. ρ for year = 2014	5087.5558 (3083.2344)	2819.2944*** (820.7124)	5127.0718 (3289.5918)	3524.2808*** (652.8267)	-0.4267 (0.2814)	-0.1826 (1.1602)	-0.2744 (0.2113)	-0.1216 (1.0374)
Average Treatment Effect	*2198.3575** (815.1592)*	*884.5791 (812.8936)*	*2554.9761** (1017.0499)*	*1967.5082** (670.1598)*	*-0.2493 (19.8910)*	*-0.1239 (0.9495)*	*-0.1595 (2.7657)*	*-0.1206 (0.9350)*
Variables used in weight determination:								
Baseline	✓	✓	✓	✓	✓	✓	✓	✓
Hospital Beds, Healthcare Expenditure and Outpatient Expenditure			✓	✓			✓	✓

*** p < 0.001, ** p < 0.01, * p < 0.05
SC: synthetic control
Single SC: Outcome-specific control group weights
Multi SC: One set of control group weights across multiple outcomes
Notes: All estimates with outcomes and control variables as levels. Approximated standard errors based on the conformal inference procedure from Chernozhukov et al. (2021) are shown in brackets.

TABLE B5. Synthetic Control approach – results for outcomes as levels

SDiD results for outcomes as levels

Time	Hospital discharges	Average length of stay
Est. ρ for year = 2005	-163.4723<br>(1511.0265)	0.0276<br>(0.6368)
Est. ρ for year = 2006	201.5341<br>(1511.0265)	0.0619<br>(0.6368)
Est. ρ for year = 2007	1343.1726<br>(1511.0265)	0.0769<br>(0.6368)
Est. ρ for year = 2008	1783.9727<br>(1511.0265)	-0.0533<br>(0.6368)
Est. ρ for year = 2009	2208.5969<br>(1511.0265)	-0.0089<br>(0.6368)
Est. ρ for year = 2010	2743.9165*<br>(1511.0265)	-0.0409<br>(0.6368)
Est. ρ for year = 2011	3541.0110**<br>(1511.0265)	-0.3735<br>(0.6368)
Est. ρ for year = 2012	4136.0368**<br>(1511.0265)	-0.4363<br>(0.6368)
Est. ρ for year = 2013	4497.4400**<br>(1511.0265)	-0.5310<br>(0.6368)
Est. ρ for year = 2014	4871.7637***<br>(1511.0265)	-0.6989<br>(0.6368)
Average treatment effect	*2772.3139**<br>*(1629.1310)*	*-0.2269*<br>*(0.5700)*

*** p < 0.001, ** p < 0.01, * p < 0.05
GDP: gross domestic product; gDRG: German (system of) diagnosis-related groups. SDID: Synthethic Difference-in-Differences
Notes: All estimates with outcomes and control variables in log form. GDP per capita and share of population aged 65 years or older used as control variables. Standard errors from placebo-evaluations in brackets.

TABLE B6. Synthetic Difference-in-differences approach – results for outcomes as levels

Appendix C – Robustness Checks

We tested the robustness of our estimates by using different sets of control units across all three methods as described in the methods section of the paper. We found that the signs and sizes of the estimates generally remained similar. Excluding all countries with comprehensive ABF reforms from our control group, independent of timing, led to mostly larger effect sizes. Using only FB countries led to volatile results because of the small sample size, with the SC approach being especially prone to high volatility. In contrast, SDiD appeared more robust to variations because it spreads weights more equally by design.

Following the critique of Rambachan and Roth (2023), we also conducted a sensitivity analysis of our DiD estimates to assess violations of the parallel trends assumption. First, the approach allowed us to judge the validity of our results based on the observed worst-case linear violation in the pre-intervention period. These were robust to roughly half the level of the maximal pre-intervention parallel trend violation. We consider this sufficient because this violation occurred several years before the intervention. Second, allowing for a non-linearity in the differential trend between Germany and the control countries that is about half the maximum observed in the pre-intervention period would render our DiD results non-significant.

For the SC approach, we estimated placebo effects for each control country as a placebo-in-space test. The change in Germany exceeded corresponding changes in all other countries. This test statistic provided a placebo p-value of 0.047 for the SC approach. Germany also outranked the placebo interventions regarding the quality of fit, as underscored by the ratio of post- vs. pre-intervention root mean square prediction error.

Placebo-in-time tests, which involve bringing the intervention forward to an earlier point in time, are generally difficult to conduct when limited data are available. Nevertheless, our approach provided credible trajectories. Germany and its (synthetic) control begin to diverge only after the gDRG system was introduced, despite having backdated it to the year 2000. Repeating our analysis with postponed intervention timing (t = 2006 and 2009) led to overall similar effect estimates as shown below. Additional results for years 2007 and 2008 are similar and availabe on request.

Due to these robustness checks and our use of different methods of analysis, the main threat to the credibility of our results remains the presence of other reforms within Germany that might explain the sudden change in hospital activity. If such reforms had an impact on hospital activity, it would not be possible to attribute the estimated effects to the introduction of the gDRG system. To rule out other external factors, two other aspects of the hospital sector must be considered in particular (Dubas-Jakóbczyk et al., 2020): hospital governance and reforms in other healthcare sectors. The former includes any reform affecting hospital capacity, management or quality governance. The latter includes reforms in primary care, long-term care or integrated care models.

In Germany, the states are responsible for hospital capacity planning. Most planning processes involve little more than revising historically derived bed numbers. In general, the algorithms used to do so employ only a limited set of parameters, such as the number of residents in a region or the frequency of hospitalizations. Even today, activity-based planning or quality requirements play a negligible role, if any. Hospital governance reforms therefore cannot explain any variation in hospital activity, especially not of the size seen in our analysis.

Regarding relationships with other sectors, a reform of the German ambulatory care payment system in 2007/08 deserves some scrutiny, particularly given that a large proportion of specialist care in Germany is provided outside of hospitals by office-based physicians. Before 2007/08, payments for ambulatory care (within the statutory health insurance system) were negotiated on a regional basis between statutory health insurers and regional associations of physicians. The overall payment made to each regional association (to be distributed among physicians in that region using other payment mechanisms) was based on capitation per insured person, covering all ambulatory services within all specialties. Simply speaking, additional services were financed by lowering the average value of every other service provided by all physicians. The reform in 2007/08 replaced this approach with a system that pays (largely) fixed values for each activity within practice-based budgets. The declared goal was to shift the morbidity risk from physicians to the statutory health insurers. This payment reform for ambulatory care had no notable direct effect on hospitals in Germany given that they almost exclusively provide inpatient services. However, changes in the provision of ambulatory

care services can also have indirect effects on hospitals, either via ambulatory care sensitive conditions (Purdy, Griffin, Salisbury, & Sharp, 2009) or by changed referral patterns. The reform to the German system of ambulatory care payment was accompanied by significant additional funding, and the little data available show no sign of decreasing activity in the ambulatory sector (see Appendix A). It is therefore unlikely that this reform could have led to the additional inpatient activity observed in our estimations.

Another event that could have led to different trends among the considered countries might be the financial crisis of 2007/08 and the subsequent European debt crisis. Although these crises affected all of the countries considered in our study, including Germany (albeit to different degrees), and we controlled for GDP, we cannot completely rule out any effect. Some countries cut their healthcare budgets substantially, and the phased introduction of the gDRG system overlaps with these cuts. However, because our results are driven more by increases in hospital activity in Germany than by decreases in the control countries, a plausible channel through which the financial crises could have caused these changes is not apparent. Furthermore, the first effects of introducing the gDRG system already become visible before the financial crisis.

DiD results gDRG introduction in 2006

	(1)	(2)	(3)	(4)	(5)
Est. ρ for year = 1	0.0561 (0.0472)	0.0465 (0.0505)	0.0118 (0.0300)	0.1071 (0.0665)	0.0719 (0.0350)
Est. ρ for year = 2	0.0881 (0.0549)	0.0687 (0.0593)	0.0432 (0.0393)	0.1415 (0.0777)	0.1180* (0.0445)
Est. ρ for year = 3	0.0969 (0.0592)	0.0738 (0.0615)	0.0646 (0.0435)	0.1650 (0.0832)	0.1422** (0.0465)
Est. ρ for year = 4	0.1161 (0.0619)	0.0832 (0.0659)	0.1224* (0.0478)	0.1774 (0.0867)	0.1742** (0.0490)
Est. ρ for year = 5	0.1358* (0.0628)	0.0976 (0.0663)	0.1353* (0.0563)	0.1940* (0.0908)	0.1972*** (0.0504)
Est. ρ for year = 6	0.1708** (0.0566)	0.1244 (0.0626)	0.1536* (0.0560)	0.2208* (0.0911)	0.2248*** (0.0520)
Est. ρ for year = 7	0.1944*** (0.0508)	0.1454* (0.0580)	0.1722** (0.0523)	0.2280* (0.0860)	0.2376*** (0.0518)
Est. ρ for year = 8	0.2218*** (0.0460)	0.1706** (0.0548)	0.1865** (0.0509)	0.2425** (0.0812)	0.2506*** (0.0483)
Est. ρ for year = 9	0.2393*** (0.0446)	0.1862** (0.0529)	0.1919** (0.0473)	0.2485** (0.0764)	0.2597*** (0.0472)
Average Treatment Effect	*0.1606** (0.0487)*	*0.1224* (0.0552)*	*0.1312** (0.0401)*	*0.2005* (0.0785)*	*0.1943*** (0.0447)*
Observations	519	467	221	382	376
Controls for:					
Baseline	✓	✓	✓	✓	✓
Hospital Beds		✓	✓	✓	✓
Private Hospital Beds			✓		
Healthcare and Outpatient Expenditure				✓	✓
Lagged Values					✓

*** p < 0.001, ** p < 0.01, * p < 0.05
Notes: All estimates include country and year fixed effects, with outcomes and control variables in log form. Robust standard errors clustered at the country level in brackets. Results for average length of stay are not provided because we do not assume the parallel trend assumption to hold.

TABLE C1. Difference-in-differences approach – Estimated impact of the gDRG introduction with treatment moved to 2006

DiD results gDRG introduction in 2009

	(1)	(2)	(3)	(4)	(5)
Est. ρ for year = 1	0.0955<br>(0.0498)	0.0659<br>(0.0520)	0.1025**<br>(0.0342)	0.1343<br>(0.0686)	0.1456**<br>(0.0403)
Est. ρ for year = 2	0.1156*<br>(0.0517)	0.0805<br>(0.0531)	0.1161*<br>(0.0431)	0.1510*<br>(0.0728)	0.1688***<br>(0.0419)
Est. ρ for year = 3	0.1516**<br>(0.0468)	0.1082*<br>(0.0506)	0.1357**<br>(0.0446)	0.1795*<br>(0.0735)	0.1965***<br>(0.0435)
Est. ρ for year = 4	0.1760***<br>(0.0418)	0.1298*<br>(0.0467)	0.1553**<br>(0.0441)	0.1889*<br>(0.0691)	0.2098***<br>(0.0436)
Est. ρ for year = 5	0.2039***<br>(0.0380)	0.1556**<br>(0.0443)	0.1705**<br>(0.0449)	0.2049**<br>(0.0649)	0.2236***<br>(0.0407)
Est. ρ for year = 6	0.2219***<br>(0.0376)	0.1715***<br>(0.0432)	0.1767**<br>(0.0436)	0.2126**<br>(0.0605)	0.2333***<br>(0.0398)
Average Treatment Effect	*0.1729***<br>(0.0409)*	*0.1300**<br>(0.0450)*	*0.1468**<br>(0.0382)*	*0.1860**<br>(0.0642)*	*0.2030***<br>(0.0393)*
Observations	519	467	221	382	376
Controls for:					
Baseline	✓	✓	✓	✓	✓
Hospital Beds		✓	✓	✓	✓
Private Hospital Beds			✓		
Healthcare and Outpatient Expenditure				✓	✓
Lagged Values					✓

*** $p < 0.001$, ** $p < 0.01$, * $p < 0.05$
Notes: All estimates include country and year fixed effects, with outcomes and control variables in log form. Robust standard errors clustered at the country level in brackets. Results for average length of stay are not provided because we do not assume the parallel trend assumption to hold.

TABLE C2. Difference-in-differences approach – Estimated impact of the gDRG introduction with treatment moved to 2009

SC results gDRG introduction in 2006

	Hospital discharges				Average length of stay			
	**Single SC**	**Multi SC**	**Single SC with Controls**	**Multi SC with Controls**	**Single SC**	**Multi SC**	**Single SC with Controls**	**Multi SC with Controls**
2006	-0.0012<br>(0.1782)	-0.0659*<br>(0.0324)	0.0137<br>(0.1840)	0.0203<br>(0.0875)	-0.0077<br>(0.0143)	-0.0064<br>(0.1068)	0.0004<br>(0.0184)	-0.0261<br>(0.0986)
2007	0.0268<br>(0.1782)	-0.0321<br>(0.0451)	0.0437<br>(0.1840)	0.0442<br>(0.0762)	0.0009<br>(0.0143)	0.0010<br>(0.0996)	0.0261<br>(0.0184)	-0.0097<br>(0.0969)
2008	0.0514<br>(0.1782)	-0.0002<br>(0.0491)	0.0703<br>(0.1840)	0.0724<br>(0.0630)	-0.0040<br>(0.0143)	-0.0003<br>(0.1047)	0.0178<br>(0.0184)	-0.0158<br>(0.1009)
2009	0.0797<br>(0.1782)	0.0174<br>(0.0468)	0.0986<br>(0.1840)	0.0715<br>(0.0374)	-0.0033<br>(0.0143)	0.0038<br>(0.1217)	0.0265<br>(0.0184)	-0.0139<br>(0.1196)
2010	0.1092<br>(0.1782)	0.0436<br>(0.0510)	0.1283<br>(0.1840)	0.0939**<br>(0.0295)	-0.0053<br>(0.0143)	0.0037<br>(0.1312)	-0.0120<br>(0.0184)	-0.0292<br>(0.1232)
2011	0.1614<br>(0.1782)	0.0897<br>(0.0619)	0.1796<br>(0.1840)	0.1384***<br>(0.0229)	-0.0167<br>(0.0143)	-0.0040<br>(0.1518)	-0.0272<br>(0.0184)	-0.0437<br>(0.1323)
2012	0.1978<br>(0.1782)	0.1030<br>(0.0542)	0.2122<br>(0.1840)	0.1555***<br>(0.0296)	-0.0034<br>(0.0143)	0.0133<br>(0.1554)	-0.0132<br>(0.0184)	-0.0300<br>(0.1321)
2013	0.2400<br>(0.1782)	0.1164*<br>(0.0472)	0.2411<br>(0.1840)	0.1661***<br>(0.0296)	-0.0075<br>(0.0143)	0.0107<br>(0.1585)	-0.0142<br>(0.0184)	-0.0330<br>(0.1304)
2014	0.2733<br>(0.1782)	0.1271**<br>(0.0417)	0.2688<br>(0.1840)	0.1763***<br>(0.0333)	-0.0171<br>(0.0143)	0.0028<br>(0.1679)	-0.0169<br>(0.0184)	-0.0408<br>(0.1385)
Average Treatment Effect	*0.1431*<br>(0.0588)*	*0.0590<br>(0.0442)*	*0.1546*<br>(0.0654)*	*0.1140**<br>(0.0394)*	*-0.0099<br>(0.7878)*	*-0.0002<br>(0.1302)*	*-0.0016<br>(0.0197)*	*-0.0286<br>(0.1192)*
Variables used in weight determination:								
Baseline	✓	✓	✓	✓	✓	✓	✓	✓
Hospital Beds, Healthcare Expenditure and Outpatient Expenditure			✓	✓			✓	✓

*** p < 0.001, ** p < 0.01, * p < 0.05
SC: synthetic control
Single SC: Outcome-specific control group weights
Multi SC: One set of control group weights across multiple outcomes
Notes: All estimates with outcomes and control variables in log form. Approximated standard errors based on the conformal inference procedure from Chernozhukov et al. (2021) are shown in brackets.

TABLE C3. Synthetic Control approach – Estimated impact of the gDRG introduction with treatment moved to 2006

SC results gDRG introduction in 2009

	Hospital discharges				Average length of stay			
	**Single SC**	**Multi SC**	**Single SC with Controls**	**Multi SC with Controls**	**Single SC**	**Multi SC**	**Single SC with Controls**	**Multi SC with Controls**
2009	0.0642 (0.1900)	0.0192 (0.0315)	0.0688 (0.1743)	0.0625* (0.0255)	-0.0005 (0.0152)	0.0088 (0.1096)	0.0233 (0.0197)	-0.0044 (0.1161)
2010	0.0934 (0.1900)	0.0452 (0.0339)	0.0944 (0.1743)	0.0847*** (0.0186)	-0.0026 (0.0152)	0.0091 (0.1184)	-0.0141 (0.0197)	-0.0179 (0.1226)
2011	0.1448 (0.1900)	0.0911* (0.0421)	0.1377 (0.1743)	0.1273*** (0.0196)	-0.0143 (0.0152)	0.0021 (0.1401)	-0.0296 (0.0197)	-0.0317 (0.1324)
2012	0.1783 (0.1900)	0.1045** (0.0347)	0.1648 (0.1743)	0.1447*** (0.0230)	-0.0018 (0.0152)	0.0197 (0.1447)	-0.0159 (0.0197)	-0.0185 (0.1332)
2013	0.2144 (0.1900)	0.1180*** (0.0323)	0.1934 (0.1743)	0.1560*** (0.0219)	-0.0061 (0.0152)	0.0172 (0.1488)	-0.0171 (0.0197)	-0.0220 (0.1314)
2014	0.2461 (0.1900)	0.1288*** (0.0282)	0.2214 (0.1743)	0.1669*** (0.0242)	-0.0160 (0.0152)	0.0096 (0.1584)	-0.0200 (0.0197)	-0.0293 (0.1398)
Average Treatment Effect	*0.1724* (0.0831)*	*0.1003*** (0.0302)*	*0.1602* (0.0665)*	*0.1335*** (0.0152)*	*-0.0105 (0.8391)*	*0.0064 (0.1316)*	*-0.0113 (0.0867)*	*-0.0223 (0.1290)*
Variables used in weight determination:								
Baseline	✓	✓	✓	✓	✓	✓	✓	✓
Hospital Beds, Healthcare Expenditure and Outpatient Expenditure			✓	✓			✓	✓

*** p < 0.001, ** p < 0.01, * p < 0.05
SC: synthetic control
Single SC: Outcome-specific control group weights
Multi SC: One set of control group weights across multiple outcomes
Notes: All estimates with outcomes and control variables in log form. Approximated standard errors based on the conformal inference procedure from Chernozhukov et al. (2021) are shown in brackets.

TABLE C4. Synthetic Control approach – Estimated impact of the gDRG introduction with treatment moved to 2009

SDiD results gDRG introduction in 2006

time	Hospital discharges	Average length of stay
2006	0.0154<br>(0.1114)	0.0022<br>(0.0895)
2007	0.0624<br>(0.1114)	0.0091<br>(0.0895)
2008	0.0834<br>(0.1114)	-0.0063<br>(0.0895)
2009	0.1096<br>(0.1114)	0.0052<br>(0.0895)
2010	0.1303<br>(0.1114)	0.0009<br>(0.0895)
2011	0.1714<br>(0.1114)	-0.0518<br>(0.0895)
2012	0.2027*<br>(0.1114)	-0.0587<br>(0.0895)
2013	0.2187*<br>(0.1114)	-0.0701<br>(0.0895)
2014	0.2304*<br>(0.1114)	-0.0916<br>(0.0895)
Average treatment effect	*0.1474*<br>*(0.1069)*	*-0.0324*<br>*(0.0880)*

*** p < 0.001, ** p < 0.01, * p < 0.05
GDP: gross domestic product; gDRG: German (system of) diagnosis-related groups. SDID: Synthethic Difference-in-Differences
Notes: All estimates with outcomes and control variables in log form. GDP per capita and share of population aged 65 years or older used as control variables. Standard errors from placebo-evaluations in brackets.

TABLE C5. Synthetic Difference-in-differences approach – Estimated impact of the gDRG introduction with treatment moved to 2006

SDiD results gDRG introduction in 2009

time	Hospital discharges	Average length of stay
2009	0.0377<br>(0.0647)	0.0117<br>(0.0618)
2010	0.0497<br>(0.0647)	0.0089<br>(0.0618)
2011	0.0926<br>(0.0647)	-0.0438<br>(0.0618)
2012	0.1295*<br>(0.0647)	-0.0480<br>(0.0618)
2013	0.1470*<br>(0.0647)	-0.0590<br>(0.0618)
2014	0.1586**<br>(0.0647)	-0.0780<br>(0.0618)
Average treatment effect	*0.1129**<br>*(0.0593)*	*-0.0362*<br>*(0.0554)*

*** p < 0.001, ** p < 0.01, * p < 0.05
GDP: gross domestic product; gDRG: German (system of) diagnosis-related groups. SDID: Synthethic Difference-in-Differences
Notes: All estimates with outcomes and control variables in log form. GDP per capita and share of population aged 65 years or older used as control variables. Standard errors from placebo-evaluations in brackets.

TABLE C6. Synthetic Difference-in-differences approach – Estimated impact of the gDRG introduction with treatment moved to 2009

DiD results without countries with CBP classification

	(1)	(2)	(3)	(4)	(5)
Est. ρ for year = 2005	0.0795<br>(0.0529)	0.0875<br>(0.0618)	0.0034<br>(0.0355)	0.1476*<br>(0.0580)	0.1095**<br>(0.0303)
Est. ρ for year = 2006	0.0922<br>(0.0586)	0.0986<br>(0.0681)	0.0194<br>(0.0493)	0.1781*<br>(0.0700)	0.1409***<br>(0.0339)
Est. ρ for year = 2007	0.1232<br>(0.0687)	0.1204<br>(0.0776)	0.0430<br>(0.0581)	0.2199*<br>(0.0805)	0.1842***<br>(0.0433)
Est. ρ for year = 2008	0.1261<br>(0.0738)	0.1193<br>(0.0789)	0.0609<br>(0.0627)	0.2503*<br>(0.0871)	0.2186***<br>(0.0473)
Est. ρ for year = 2009	0.1551<br>(0.0773)	0.1394<br>(0.0839)	0.1500*<br>(0.0638)	0.2728**<br>(0.0905)	0.2648***<br>(0.0499)
Est. ρ for year = 2010	0.1755*<br>(0.0822)	0.1535<br>(0.0856)	0.1637*<br>(0.0692)	0.2968**<br>(0.0974)	0.2922***<br>(0.0520)
Est. ρ for year = 2011	0.2105*<br>(0.0783)	0.1793*<br>(0.0815)	0.2013*<br>(0.0644)	0.3296**<br>(0.0987)	0.3209***<br>(0.0555)
Est. ρ for year = 2012	0.2328**<br>(0.0715)	0.1985*<br>(0.0750)	0.2284**<br>(0.0590)	0.3324**<br>(0.0942)	0.3335***<br>(0.0595)
Est. ρ for year = 2013	0.2531**<br>(0.0674)	0.2169**<br>(0.0716)	0.2486**<br>(0.0560)	0.3362**<br>(0.0914)	0.3330***<br>(0.0610)
Est. ρ for year = 2014	0.2719***<br>(0.0656)	0.2333**<br>(0.0689)	0.2614***<br>(0.0532)	0.3435**<br>(0.0870)	0.3416***<br>(0.0595)
Average Treatment Effect	*0.1869**<br>(0.0607)*	*0.1661*<br>(0.0682)*	*0.1574**<br>(0.0481)*	*0.2777**<br>(0.0835)*	*0.2589***<br>(0.0482)*
Observations	343	301	147	243	238
Controls for:					
Baseline	✓	✓	✓	✓	✓
Hospital Beds		✓	✓	✓	✓
Private Hospital Beds			✓		
Healthcare and Outpatient Expenditure				✓	✓
Lagged Values					✓

*** p < 0.001, ** p < 0.01, * p < 0.05
Notes: All estimates include country and year fixed effects, with outcomes and control variables in log form. Robust standard errors clustered at the country level in brackets. Results for average length of stay are not provided because we do not assume the parallel trend assumption to hold.

Prefit not tested, thus bad prefits might affect results.

TABLE C7. Difference-in-differences approach – Estimated impact of the gDRG introduction without countries with Case Based Payments classification as controls

DiD results with fixed budget only classification

	(1)	(2)	(3)	(4)	(5)
Est. ρ for year = 2005	-0.0403<br>(0.0561)	0.0189<br>(0.0412)	0.0171<br>(0.0352)	0.0403<br>(0.0406)	-0.0440<br>(0.0404)
Est. ρ for year = 2006	-0.0694<br>(0.0609)	-0.0059<br>(0.0404)	0.0035<br>(0.0402)	0.0810<br>(0.0370)	-0.0432<br>(0.0472)
Est. ρ for year = 2007	-0.0778<br>(0.0603)	-0.0493<br>(0.0354)	-0.0212<br>(0.0471)	0.1095<br>(0.0564)	-0.0305<br>(0.0632)
Est. ρ for year = 2008	-0.1309<br>(0.0811)	-0.0951<br>(0.0409)	-0.0516<br>(0.0735)	0.1138<br>(0.0554)	-0.0560<br>(0.0772)
Est. ρ for year = 2009	-0.1313<br>(0.0969)	-0.0314<br>(0.0315)	0.0165<br>(0.0634)	0.1249<br>(0.0550)	-0.0206<br>(0.0817)
Est. ρ for year = 2010	-0.1376<br>(0.1135)	-0.0620<br>(0.0314)	-0.0450<br>(0.0768)	0.1348<br>(0.0614)	0.0094<br>(0.0722)
Est. ρ for year = 2011	-0.0870<br>(0.1314)	-0.0375<br>(0.0360)	-0.0506<br>(0.0759)	0.1373<br>(0.0686)	0.0457<br>(0.1043)
Est. ρ for year = 2012	-0.0250<br>(0.1279)	-5.21e-5<br>(0.0374)	-0.0278<br>(0.0574)	0.1683*<br>(0.0476)	0.1344<br>(0.0909)
Est. ρ for year = 2013	0.0312<br>(0.1176)	0.0391<br>(0.0329)	-0.0047<br>(0.0414)	0.1980**<br>(0.0407)	0.1804<br>(0.0861)
Est. ρ for year = 2014	0.0754<br>(0.1131)	0.0780*<br>(0.0296)	0.0183<br>(0.0345)	0.2150**<br>(0.0430)	0.2108*<br>(0.0832)
Average Treatment Effect	*-0.0175*<br>*(0.0753)*	*0.0256*<br>*(0.0374)*	*0.0265*<br>*(0.0249)*	*0.1012***<br>*(0.0264)*	*-0.0036*<br>*(0.0527)*
Observations	149	112	63	86	83
Controls for:					
Baseline	✓	✓	✓	✓	✓
Hospital Beds		✓	✓	✓	✓
Private Hospital Beds			✓		
Healthcare and Outpatient Expenditure				✓	✓
Lagged Values					✓

*** p < 0.001, ** p < 0.01, * p < 0.05
Notes: All estimates include country and year fixed effects, with outcomes and control variables in log form. Robust standard errors clustered at the country level in brackets. Results for average length of stay are not provided because we do not assume the parallel trend assumption to hold.

Prefit not tested, thus bad prefits might affect results.

TABLE C8. Difference-in-differences approach – Estimated impact of the gDRG introduction with Fixed Budget classification countries as controls

DiD results with alternative classification

	(1)	(2)	(3)	(4)	(5)
Est. ρ for year = 2005	0.0979 (0.0497)	0.0960 (0.0568)	0.0126 (0.0229)	0.1386* (0.0638)	0.0772 (0.0385)
Est. ρ for year = 2006	0.1123 (0.0588)	0.1102 (0.0661)	0.0227 (0.0318)	0.1721* (0.0754)	0.1014* (0.0434)
Est. ρ for year = 2007	0.1528* (0.0689)	0.1420 (0.0775)	0.0569 (0.0415)	0.2179* (0.0877)	0.1549** (0.0540)
Est. ρ for year = 2008	0.1604* (0.0765)	0.1461 (0.0837)	0.0744 (0.0463)	0.2456* (0.0939)	0.1798** (0.0576)
Est. ρ for year = 2009	0.1773* (0.0815)	0.1530 (0.0922)	0.1184* (0.0506)	0.2552* (0.0971)	0.2119** (0.0605)
Est. ρ for year = 2010	0.1938* (0.0821)	0.1657 (0.0911)	0.1328* (0.0571)	0.2754* (0.0997)	0.2353*** (0.0601)
Est. ρ for year = 2011	0.2197** (0.0729)	0.1828* (0.0836)	0.1446* (0.0571)	0.2923** (0.0981)	0.2654*** (0.0609)
Est. ρ for year = 2012	0.2394** (0.0645)	0.1995* (0.0753)	0.1624** (0.0527)	0.2961** (0.0925)	0.2765*** (0.0600)
Est. ρ for year = 2013	0.2565*** (0.0590)	0.2141** (0.0700)	0.1738** (0.0513)	0.3018** (0.0886)	0.2797*** (0.0548)
Est. ρ for year = 2014	0.2697*** (0.0549)	0.2247** (0.0652)	0.1793** (0.0462)	0.3040** (0.0830)	0.2878*** (0.0532)
Average Treatment Effect	*0.1998** (0.0605)*	*0.1719* (0.0711)*	*0.1185** (0.0400)*	*0.2547** (0.0849)*	*0.2131*** (0.0513)*
Observations	475	423	242	355	349
Controls for:					
Baseline	✓	✓	✓	✓	✓
Hospital Beds		✓	✓	✓	✓
Private Hospital Beds			✓		
Healthcare and Outpatient Expenditure				✓	✓
Lagged Values					✓

*** $p < 0.001$, ** $p < 0.01$, * $p < 0.05$
Notes: All estimates include country and year fixed effects, with outcomes and control variables in log form. Robust standard errors clustered at the country level in brackets. Results for average length of stay are not provided because we do not assume the parallel trend assumption to hold.

Prefit not tested, thus bad prefits might affect results.

TABLE C9. Difference-in-differences approach – Estimated impact of the gDRG introduction with alternative classification approach

SC results without countries with CBP

	Hospital discharges				Average length of stay			
	**Single SC**	**Multi SC**	**Single SC with Controls**	**Multi SC with Controls**	**Single SC**	**Multi SC**	**Single SC with Controls**	**Multi SC with Controls**
Est. ρ for year = 2005	0.0581 (0.2565)	0.0679 (0.2267)	0.2213 (0.4525)	0.0630*** (0.0000)	0.0583 (0.0525)	0.1096 (0.0933)	0.0892 (0.1541)	0.0953*** (0.0000)
Est. ρ for year = 2006	0.0846 (0.2565)	0.0675 (0.2535)	0.2428 (0.4525)	0.0598*** (0.0000)	0.0655 (0.0525)	0.1343* (0.0635)	0.1128 (0.1541)	0.1221*** (0.0000)
Est. ρ for year = 2007	0.1403 (0.2565)	0.1222 (0.2531)	0.3024 (0.4525)	0.1127*** (0.0000)	0.0607 (0.0525)	0.1211** (0.0466)	0.1035 (0.1541)	0.1119*** (0.0000)
Est. ρ for year = 2008	0.1734 (0.2565)	0.1452 (0.2551)	0.3435 (0.4525)	0.1360*** (0.0000)	0.0626 (0.0525)	0.1368*** (0.0239)	0.1176 (0.1541)	0.1284*** (0.0000)
Est. ρ for year = 2009	0.2033 (0.2565)	0.1414 (0.2867)	0.3768 (0.4525)	0.1304*** (0.0000)	0.0588 (0.0525)	0.1651*** (0.0089)	0.1419 (0.1541)	0.1582*** (0.0000)
Est. ρ for year = 2010	0.2322 (0.2565)	0.1620 (0.3022)	0.4152 (0.4525)	0.1494*** (0.0000)	0.0547 (0.0525)	0.1820*** (0.0245)	0.1552 (0.1541)	0.1749*** (0.0000)
Est. ρ for year = 2011	0.2777 (0.2565)	0.1987 (0.3282)	0.4805 (0.4525)	0.1842*** (0.0000)	0.0399 (0.0525)	0.2005** (0.0643)	0.1696 (0.1541)	0.1951*** (0.0000)
Est. ρ for year = 2012	0.2998 (0.2565)	0.2126 (0.3343)	0.5151 (0.4525)	0.1975*** (0.0000)	0.0444 (0.0525)	0.2320* (0.1070)	0.1992 (0.1541)	0.2296*** (0.0000)
Est. ρ for year = 2013	0.3271 (0.2565)	0.2289 (0.3446)	0.5535 (0.4525)	0.2129*** (0.0000)	0.0453 (0.0525)	0.2341* (0.1184)	0.2021 (0.1541)	0.2329*** (0.0000)
Est. ρ for year = 2014	0.3425 (0.2565)	0.2402 (0.3610)	0.5864 (0.4525)	0.2230*** (0.0000)	0.0403 (0.0525)	0.2358 (0.1367)	0.2042 (0.1541)	0.2364*** (0.0000)
Average Treatment Effect	*0.2295*** (0.0000)*	*0.1810 (0.2912)*	*0.4234* (0.1508)*	*0.1691*** (0.0000)*	*0.0470 (3.7488)*	*0.1709*** (0.0276)*	*0.1453 (11.5927)*	*0.1646*** (0.0000)*
Variables used in weight determination:								
Baseline	✓	✓	✓	✓	✓	✓	✓	✓
Hospital Beds, Healthcare Expenditure and Outpatient Expenditure			✓	✓			✓	✓

*** p < 0.001, ** p < 0.01, * p < 0.05
SC: synthetic control
Single SC: Outcome-specific control group weights
Multi SC: One set of control group weights across multiple outcomes
Notes: All estimates with outcomes and control variables in log form. Approximated standard errors based on the conformal inference procedure from Chernozhukov et al. (2021) are shown in brackets.

Prefit not tested, thus bad prefits might affect results.

TABLE C10. Synthetic control approach – Estimated impact of the gDRG introduction without countries with Case Based Payment classification as controls

SC results with alternative classification

	Hospital discharges				Average length of stay			
	**Single SC**	**Multi SC**	**Single SC with Controls**	**Multi SC with Controls**	**Single SC**	**Multi SC**	**Single SC with Controls**	**Multi SC with Controls**
Est. ρ for year = 2005	0.0086 (0.1806)	0.0023 (0.1035)	-0.0029 (0.1692)	-0.0843* (0.0363)	-0.0204 (0.0199)	-0.0388 (0.0970)	-0.0100 (0.0165)	-0.0132 (0.1111)
Est. ρ for year = 2006	0.0139 (0.1806)	0.0165 (0.0993)	0.0029 (0.1692)	-0.0695 (0.0358)	-0.0038 (0.0199)	-0.0333 (0.1029)	-0.0093 (0.0165)	-0.0084 (0.1187)
Est. ρ for year = 2007	0.0457 (0.1806)	0.0411 (0.0868)	0.0304 (0.1692)	-0.0361 (0.0476)	0.0221 (0.0199)	-0.0168 (0.1021)	-0.0003 (0.0165)	-0.0007 (0.1108)
Est. ρ for year = 2008	0.0731 (0.1806)	0.0703 (0.0725)	0.0561 (0.1692)	-0.0041 (0.0512)	0.0137 (0.0199)	-0.0231 (0.1048)	-0.0059 (0.0165)	-0.0024 (0.1192)
Est. ρ for year = 2009	0.1021 (0.1806)	0.0708 (0.0438)	0.0873 (0.1692)	0.0140 (0.0493)	0.0217 (0.0199)	-0.0225 (0.1230)	-0.0059 (0.0165)	0.0011 (0.1421)
Est. ρ for year = 2010	0.1327 (0.1806)	0.0939** (0.0349)	0.1183 (0.1692)	0.0404 (0.0531)	-0.0172 (0.0199)	-0.0378 (0.1237)	-0.0084 (0.0165)	0.0006 (0.1539)
Est. ρ for year = 2011	0.1856 (0.1806)	0.1399*** (0.0246)	0.1713 (0.1692)	0.0868 (0.0668)	-0.0326 (0.0199)	-0.0526 (0.1318)	-0.0205 (0.0165)	-0.0077 (0.1714)
Est. ρ for year = 2012	0.2195 (0.1806)	0.1563*** (0.0331)	0.2042 (0.1692)	0.1002 (0.0599)	-0.0183 (0.0199)	-0.0385 (0.1303)	-0.0080 (0.0165)	0.0091 (0.1774)
Est. ρ for year = 2013	0.2481 (0.1806)	0.1666*** (0.0336)	0.2336 (0.1692)	0.1135* (0.0559)	-0.0192 (0.0199)	-0.0413 (0.1284)	-0.0123 (0.0165)	0.0063 (0.1772)
Est. ρ for year = 2014	0.2753 (0.1806)	0.1763*** (0.0381)	0.2656 (0.1692)	0.1243* (0.0500)	-0.0221 (0.0199)	-0.0496 (0.1364)	-0.0223 (0.0165)	-0.0019 (0.1867)
Average Treatment Effect	*0.1457** (0.0558)*	*0.1033* (0.0513)*	*0.1319** (0.0489)*	*0.0428 (0.0472)*	*-0.0077 (0.1134)*	*-0.0370 (0.1182)*	*-0.0128 (1.0180)*	*-0.0043 (0.1464)*
Variables used in weight determination:								
Baseline	✓	✓	✓	✓	✓	✓	✓	✓
Hospital Beds, Healthcare Expenditure and Outpatient Expenditure			✓	✓			✓	✓

*** p < 0.001, ** p < 0.01, * p < 0.05
SC: synthetic control
Single SC: Outcome-specific control group weights
Multi SC: One set of control group weights across multiple outcomes
Notes: All estimates with outcomes and control variables in log form. Approximated standard errors based on the conformal inference procedure from Chernozhukov et al. (2021) are shown in brackets.

Prefit not tested, thus bad prefits might affect results.

TABLE C11. Synthetic control approach – Estimated impact of the gDRG introduction with alternative classification

SDiD results without countries with CBP classification

time	Hospital discharges	Average length of stay
Est. ρ for year = 2005	-0.0127<br>(0.0682)	-0.0455<br>(0.0725)
Est. ρ for year = 2006	-0.0007<br>(0.0682)	-0.0541<br>(0.0725)
Est. ρ for year = 2007	0.0289<br>(0.0682)	-0.0630<br>(0.0725)
Est. ρ for year = 2008	0.0412<br>(0.0682)	-0.0935<br>(0.0725)
Est. ρ for year = 2009	0.0693<br>(0.0682)	-0.1050<br>(0.0725)
Est. ρ for year = 2010	0.0878<br>(0.0682)	-0.0841<br>(0.0725)
Est. ρ for year = 2011	0.1377*<br>(0.0682)	-0.1331*<br>(0.0725)
Est. ρ for year = 2012	0.1697**<br>(0.0682)	-0.1573*<br>(0.0725)
Est. ρ for year = 2013	0.1848**<br>(0.0682)	-0.1635*<br>(0.0725)
Est. ρ for year = 2014	0.1917**<br>(0.0682)	-0.1833**<br>(0.0725)
Average treatment effect	*0.1030*<br>*(0.0728)*	*-0.1176*<br>*(0.0751)*

*** $p < 0.001$, ** $p < 0.01$, * $p < 0.05$
GDP: gross domestic product; gDRG: German (system of) diagnosis-related groups. SDID: Synthethic Difference-in-Differences
Notes: All estimates with outcomes and control variables in log form. GDP per capita and share of population aged 65 years or older used as control variables. Standard errors from placebo-evaluations in brackets.

Prefit not tested, thus bad prefits might affect results.

TABLE C12. Synthetic difference-in-differences approach – *Estimated impact of the gDRG introduction without countries with Case Based Payment classification*

SDiD results with alternative classification

time	Hospital discharges	Average length of stay
Est. ρ for year = 2005	0.0272<br>(0.1039)	-0.0248<br>(0.0956)
Est. ρ for year = 2006	0.0329<br>(0.1039)	-0.0302<br>(0.0956)
Est. ρ for year = 2007	0.0704<br>(0.1039)	-0.0320<br>(0.0956)
Est. ρ for year = 2008	0.0817<br>(0.1039)	-0.0504<br>(0.0956)
Est. ρ for year = 2009	0.0901<br>(0.1039)	-0.0496<br>(0.0956)
Est. ρ for year = 2010	0.1084<br>(0.1039)	-0.0552<br>(0.0956)
Est. ρ for year = 2011	0.1469<br>(0.1039)	-0.0691<br>(0.0956)
Est. ρ for year = 2012	0.1747*<br>(0.1039)	-0.0770<br>(0.0956)
Est. ρ for year = 2013	0.1885*<br>(0.1039)	-0.0872<br>(0.0956)
Est. ρ for year = 2014	0.1975*<br>(0.1039)	-0.1106<br>(0.0956)
Average treatment effect	*0.1210*<br>*(0.0951)*	*-0.0638*<br>*(0.0958)*

*** p < 0.001, ** p < 0.01, * p < 0.05
GDP: gross domestic product; gDRG: German (system of) diagnosis-related groups. SDID: Synthethic Difference-in-Differences
Notes: All estimates with outcomes and control variables in log form. GDP per capita and share of population aged 65 years or older used as control variables. Standard errors from placebo-evaluations in brackets.

Prefit not tested, thus bad prefits might affect results.

TABLE C13. Synthetic difference-in-differences approach – Estimated impact of the gDRG introduction with alternative classification

Deviation from parallel trends

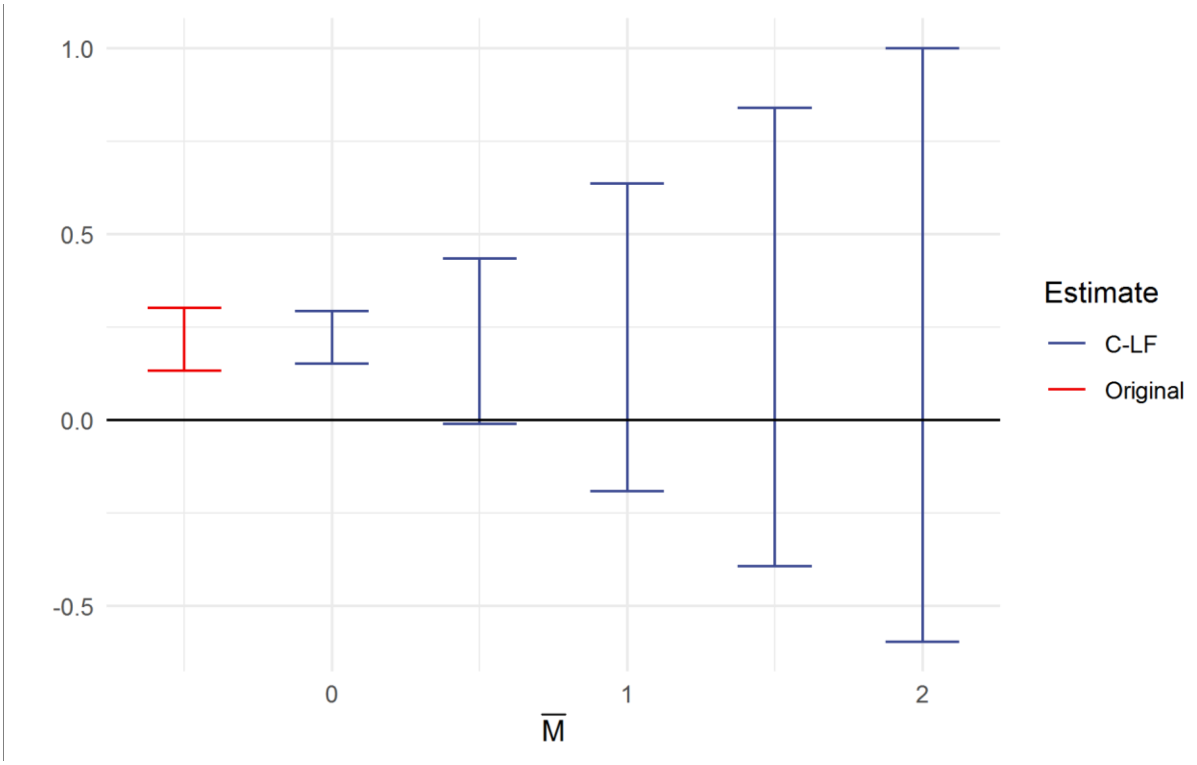

FIGURE C1. Sensitivity analysis for parallel trends – number of hospital discharges.

Notes: Estimate for t = 2014. See Rambachan and Roth (2021) for more information regarding setup and interpretation.

Deviation from linear trends

lb	ub	method	Delta	Mbar
0.1515	0.2525	C-LF	DeltaSDRM	0.0000
-0.0909	0.5152	C-LF	DeltaSDRM	0.5000
-0.3737	0.7980	C-LF	DeltaSDRM	1.0000
-0.6364	1.0000	C-LF	DeltaSDRM	1.5000
-0.9394	1.0000	C-LF	DeltaSDRM	2.0000

Notes: Estimate for t = 2014. See Rambachan and Roth (2021) for more information regarding setup and interpretation.

TABLE C14. Sensitivity for deviation from linear trend – number of hospital discharges.

.

Placebo-in-space test for hospital discharges

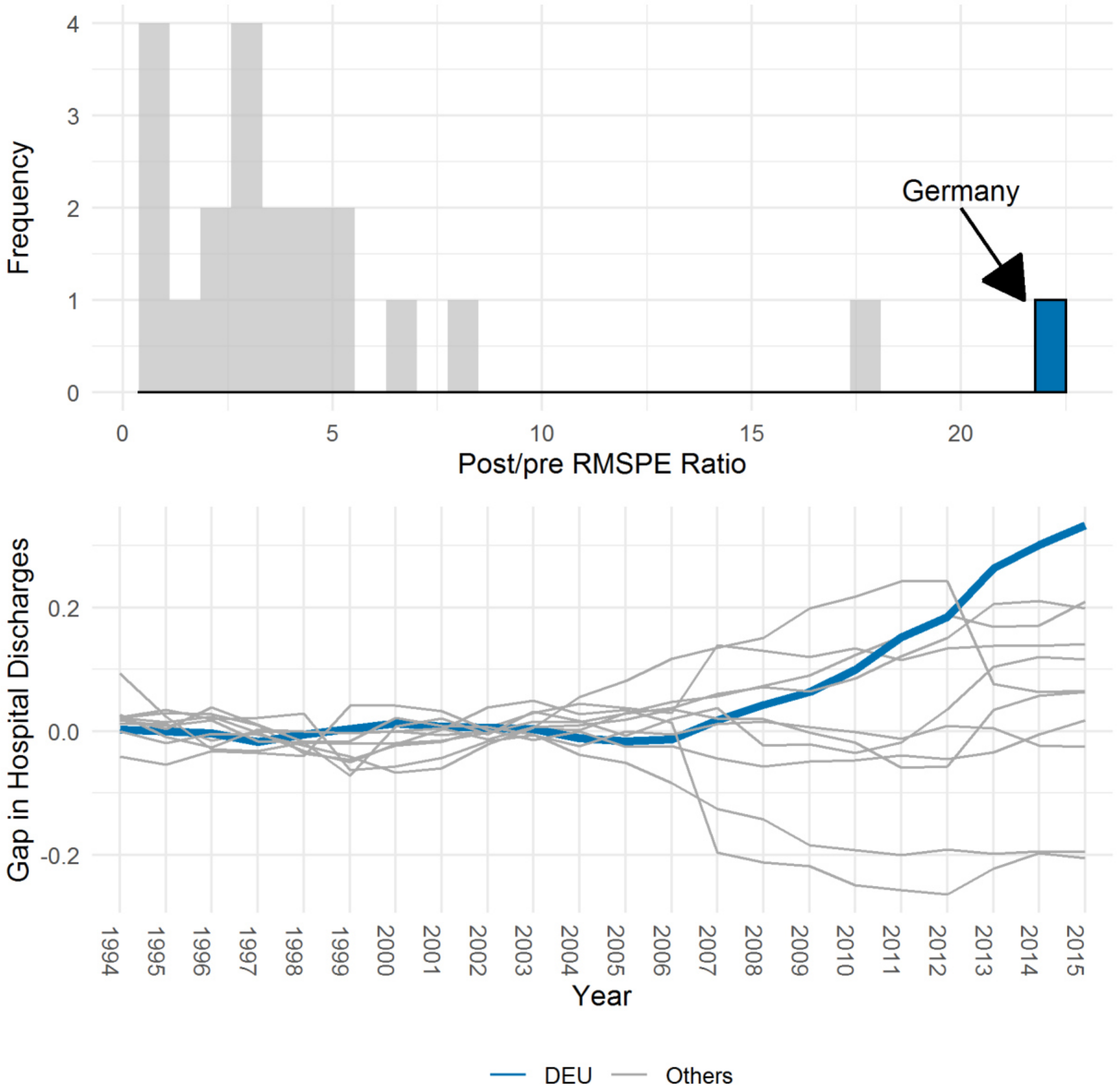

FIGURE C2. Placebo-in-space test for number of hospital discharges.

Notes: Only countries with not more than five times Germany's pre-treatment fit (RMSPE = root mean squared prediction error) are plotted below.

Placebo-in-time for hospital discharges

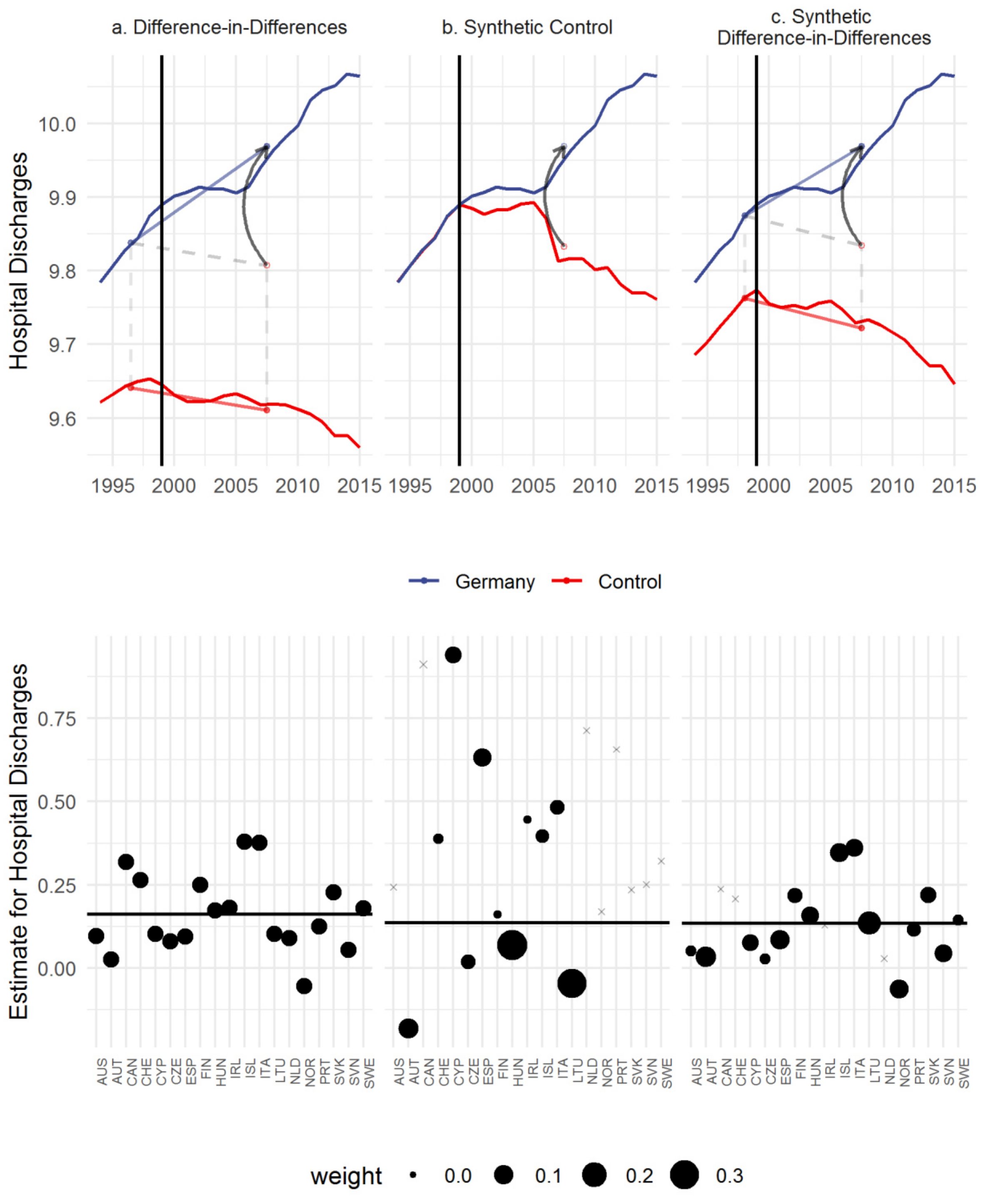

FIGURE C3. Placebo-in-time for number of hospital discharges. Treatment assigned to t=2000.

1. References

Dubas-Jakóbczyk, K., Albreht, T., Behmane, D., Bryndova, L., Dimova, A., Džakula, A., . . . Quentin, W. (2020). Hospital reforms in 11 Central and Eastern European countries between 2008 and 2019: A comparative analysis. *Health Policy*, *124*(4), 368–379. https://doi.org/10.1016/j.healthpol.2020.02.003

Purdy, S., Griffin, T., Salisbury, C., & Sharp, D. (2009). Ambulatory care sensitive conditions: Terminology and disease coding need to be more specific to aid policy makers and clinicians. *Public Health*, *123*(2), 169–173. https://doi.org/10.1016/j.puhe.2008.11.001

Rambachan, A., & Roth, J. (2023). A More Credible Approach to Parallel Trends. *Review of Economic Studies*. (rdad018).
