# Supplementary material for: Country-level effects of diagnosis-related groups: evidence from Germany’s comprehensive reform of hospital payments
Source: Eur J Health Econ. 2023 Dec 5;25(6):1013–30. doi: 10.1007/s10198-023-01645-z (PMC11283398; doi:10.1007/s10198-023-01645-z)
Supplement: Supplementary file 2 — Supplementary file2 (PDF 678 KB) [file 10198_2023_1645_MOESM2_ESM.pdf]

Supporting Information

Country-level effects of diagnosis-related groups: Evidence from Germany's comprehensive reform of hospital payments

Robert Messerle and Jonas Schreyögg[1]

Hamburg Center for Health Economics, University of Hamburg, Esplanade 36, 20354, Hamburg, Germany

In: European Journal of Health Economics

[1]Correspondence to Jonas Schreyögg, University of Hamburg, Esplanade 36, 20354, Hamburg, Germany.
Email: jonas.schreyoegg@uni-hamburg.de, Phone: +49 42838-8041

Supporting Information

Hospital payment scheme classification

To investigate the effects of introducing the gDRG system, we constructed – analogously to previous research [1, 2] – a control data set describing the main hospital payment schemes in selected OECD and EU member states from 1994 to 2015. We included data from all European OECD countries and EU member states at the time of the reform, as well as from Australia, Canada and New Zealand. This sample therefore comprised data from high-income countries with generally comparable levels of healthcare provision and served as a starting point for constructing a suitable control group. This appendix provides more details about the classification and the underlying sources.

General classification

First, to classify hospital payment schemes, we created two basic categories with opposite incentive structures: fixed budgets (FBs) and case-based payments (CBP). We classified a country as using FBs in a given year if global budgets or block grants were the main form of hospital funding. In such cases, hospital revenue is determined mostly in advance based on provider characteristics like hospital size or the range of care provided. We classified a country as using CBP if hospitals were paid mainly according to the characteristics of the patients they admitted, for example case-based payments following a DRG classification.

A major challenge was that many countries changed their hospital payment schemes only gradually or partially. As a result, CBP often only affected a fraction of hospital budgets (e.g., in Denmark), was limited to certain hospitals or regions (e.g., in Finland and Sweden) or was used for budgeting but not for actual billing processes (e.g., in Ireland). We argue that, in such cases, the change in hospital incentive structures is considerably weaker, at least at the aggregate level used in our analysis. The incentive structure in payment systems based on comprehensive CBP differs from that in systems in which CBP is one of many payment methods. Indeed, some countries might have used a mixed approach precisely in order to balance the incentive structures of different payment schemes.

To incorporate this line of thought into our classification, we introduced a mixed-funding category. We narrowed the definition of CBP to systems in which CBP was the main way to pay for hospital care and applied to the majority of acute care hospitals nationwide. We classified systems that used CBP only partially or mainly for budgeting purposes as using mixed funding. We also assigned systems with fee-for-service payments within global budgets to the mixed category.

A combination of CBP with a global budget for healthcare expenditure or an overall budget for hospital care did not necessarily lead to a classification as a mixed funding system. For example, in Hungary, DRG-based payments are used for billing purposes but the price of a DRG is not fixed because there is a global budget [3]. Increased

hospital activity therefore leads to lower prices overall. However, the incentive structure of CBP is still in place because individual hospitals cannot influence the activity of other hospitals.

Inevitably, our classifications are based on judgement and therefore cannot be purely objective. Most countries in our sample used several methods simultaneously, and the relative importance of individual schemes was often unclear. Moreover, when a payment reform is implemented gradually, it is difficult to define a point in time at which it begins. We therefore included alternative classifications for some countries in our robustness checks.

The main source of information for our classifications was the Health System in Transition series (HiT) of the European Observatory on Health Systems and Policies and the OECD Health Systems Characteristics Survey [4–6]. We primarily screened the "Payment mechanisms" section of the HiT series and used additional sources in cases where this section lacked the necessary information. Country-specific literature is referenced in table S1.

Country	Remarks and Additional Data Sources	Main Classification	In CG	Alternative classification	In CG
**Australia**	Hospitals were mainly financed through prospective global budgets. In 1993, Victoria and South Australia started adopting case-mix DRG systems for budget allocation. Nationwide implementation started in 2012. Since 2014 these have been used for billing purposes, and before this mainly for budgeting purposes. [7, 8]	Mixed 1994 - 2013<br>CBP 2014 - 2015	x		
**Austria**	Hospitals were mainly financed through prospective global budgets. In 1997, a DRG-based system called 'Leistungsorientierte Krankenhausfinanzierung' (LKF) was introduced. Austria uses this DRG-like system on a nationwide basis (for >70% of revenue); regional adjustments are possible (on average 30%) and the shares vary widely among the states from approx. 15-50%, leading to substantial price variation. [9]	FB 1994-1996<br>CBP 1997-2015	x	FB 1994-1996<br>Mixed 1997-2015	x
**Belgium**	Hospitals are financed by complex budget allocations consisting of several items. Before 2001 this budget distribution was based partly on cost comparison. DRGs were introduced in 1994, but have been used for some aspects of budget allocation only since 2002. There are no direct DRG payments. [10, 11]	FB 1994-2001<br>Mixed 2002-2015	x	FB 1994-2015	x
**Canada**	Payments are generally based on the previous year's allocation adjusted for	FB 1994-2015	x	FB 1994-2009	x

	inflation and budget growth. In 2010, CBP was partially introduced in British Columbia. The CBP program re-directed up to 20% of global budget funding to a case-mix-adjusted per case payment, replacing a portion of funding from the global budget. [12–14]			Mixed 2010-2015	
**Cyprus**	Hospitals are financed through historic global budgets. FFS only in private sector; DRGs introduced in 2019. [15]	FB 1994-2015	x		
**Czech Republic**	From 1993 to mid-1997 an FFS system was partially used for hospital reimbursement. Afterwards a prospective global budget was the main source of financing for most years. The share of hospital care paid through DRG-based case payments gradually increased from 2007. In 2012 it was the main payment mechanism for hospitals. In 2015, the Czech Republic stopped financing hospital care based on DRGs completely and returned temporarily to flat fees. [16, 17]	Mixed 1994-1996<br>FB 1997-2011, 2015<br>CBP 2012-2014	x	Mixed 1994-2011<br>FB 2015<br>CBP 2012-2014	x
**Denmark**	In Denmark, hospital budgets are partially determined based on CBP, while they also depend on annually negotiated global budgets. In 1999, after the introduction of DRGs, 10% of the budget was allocated by CBP, and in 2008 the mandatory share was set to 50%. Since 2015, value based procurement has become more important. [18, 19]<br>Not in control group for comprehensive hospital reform.	FB 1994-1999<br>Mixed 2000-2015		FB 1994-1999<br>Mixed 2000-2007<br>CBP 2008-2015	
**Estonia**	Fee-for-service and per diem system within a global budget was used until 2003. In 2003, the DRG system was introduced for case grouping. From 2004, the NordDRG system became operational in combination with other payment methods. The proportion of DRG payments was gradually raised from 10% in 2004 to 70% in 2009. [20–23]	Mixed 1994-2007<br>CBP 2008-2015			
**Finland**	Region-specific budgets are used to finance hospitals. Often NordDRG based methods are used for invoicing to municipalities. [24–26]	Mixed 1994-2015	x		
**France**	Global budgets were mainly used for hospital financing before 2004, at least for public hospitals. DRGs were introduced in 2004/2005. In 2010, 56% of total hospital expenditure was covered by DRG-based payments. [10, 27, 28]	FB 1994-2003<br>CBP 2004-2015		FB 1994-2003<br>Mixed 2004-2015	x

**Germany**	From 1993, payments were based on prospective fixed budgets (and before that on full cost compensation). In 2004, the change to the DRG system took place. [29]	FB 1994-2003<br><br>CBP 2004-2015			
**Greece**	Until 2010, hospitals were paid within a retrospective per diem system under a global budget. The government periodically compensated deficits. The German DRG system was implemented from 2011. [30, 31]	Mixed 1994-2010<br><br>CBP 2011-2015	x		
**Hungary**	DRGs were introduced as early as 1993. However, DRGs are not used as fixed payment units. Instead prices of DRGs vary with budget utilization. [3, 32, 33]	CBP 1994-2015	x	Mixed 1994-2015	x
**Iceland**	Since the 1980s, Iceland has mainly used a fixed budget. The NordDRG System has been in place in Iceland since 2001 but DRGs are mainly used for internal distribution of the budget to departments within hospitals. [24]	FB 1994-2015	x		
**Ireland**	Ireland predominately uses historic budgets for hospital financing. In 2001, a full-phased implementation of CBP for financing was announced. However, in 2021 budget allocation was still only partially based on CBP for some hospitals. [34–36]	FB 1994-2015	x	FB 1994-2000<br><br>CBP 2001-2015	
**Italy**	Hospitals were predominately paid based on fixed budgets. DRGs were introduced in 1995, but the degree of use varies strongly according to region. Moreover, budgets for specific services (e.g. emergency services, oncology treatments, teaching activities) are not based on DRG tariffs but are paid on the basis of global budgets. Focus on CBP has faded recently. [37–39]	FB 1994<br><br>Mixed 1995-2015	x	FB 1994<br><br>CBP 1995-2015	
**Latvia**	Until 2010 Latvia used a mix of case-based and per diem payments; afterwards, fixed budgets were implemented. DRGs were implemented starting in 2014, but their role is limited. [40, 41]	Mixed 1994-2009, 2015<br><br>FB 2010-2014	x		
**Lithuania**	Before 2012, hospitals were paid for admitted patients according to major specialty. DRG-based payments were introduced in 2012. [42]	Mixed 1995-2011<br><br>CBP 2012-2015	x		
**Luxembourg**	Hospital are funded by global budgets. [43]	FB 1994-2015	x		
**Malta**	Hospital are funded by global budgets. [44]	FB 1994-2015	x		
**New Zealand**	Public hospitals receive an annual budget. [45]	FB 1994-2015	x		
**Netherlands**	Hospitals were predominately reimbursed by fixed budgets. In 2001, there was a	FB 1994-2000	x	FB 1994-2000	

	change to FFS and in 2005 the first adoption of a DRG-like system (DBCs); by 2008 about 20% of hospital costs were paid through DBCs. In 2012, fundamental changes were implemented and stronger emphasis placed on DBCs. [46–48]	Mixed 2001-2011<br>CBP 2012-2015		Mixed 2001-2004<br>CBP 2005-2015	
**Norway**	Hospital financing changed in 1997 from fixed budgets to a mixed financing system with budgets and case-based payments. [24, 49].	FB 1994-1996<br>Mixed 1997-2015	x		
**Poland**	Until 1999 historical budgets were used for hospital funding; afterwards, hospital payments were based on an activity-based catalog. DRGs were implemented as a nationwide hospital financing system in 2009. However, for some parts of hospital care they are only used to calculate biannual lump sum payments.<br>[1, 50, 51]	FB 1994-1999<br>Mixed 2000-2008<br>CBP 2009-2015		FB 1994-1999<br>Mixed 2000-2015	x
**Portugal**	The Portuguese NHS funds inpatient care through global budgets. Between 1997 and 2002 funding through DRGs gradually increased. Since 2003 it has represented around 75–85% of NHS hospitals' inpatient budget. DRGs are used for budget allocation but not payments. [52]	FB 1994-1996<br>Mixed 1997-2015	x	FB 1994-1996<br>CBP 1997-2015	
**Slovak Republic**	From 1993 to 2000, several distinct payment mechanisms followed in short succession. Since 2001, hospitals have been funded through several channels: case payments grouped by departments and hospitals, fee-for-service and per diem payments. DRG implementation started 2016. [53]	Mixed 1994-1999<br>FB 2000-2001<br>Mixed 2002-2015	x		
**Slovenia**	Hospital payments are based on provider budgets. Since 2003, DRG case payments have been gradually introduced into budget negotiations. [54]	FB 1994-2002<br>Mixed 2003-2015	x	FB 1994-2002<br>CBP 2003-2015	
**Spain**	Hospitals are primarily financed by global budgets. Regional (especially Catalonia) use of DRGs for budget allocation. DRGs are also used for regional balance payments. [55]	FB 1994-2015	x		
**Sweden**	Before 2002, Sweden used fixed budgets as the main hospital payment scheme. In 2002, hospitals introduced case payments based on DRGs as a partial aspect of payment. Budget allocation methods differ among regions, and global budgets still play a major role. [56]	FB 1994-2001<br>Mixed 2002-2015	x	FB 1994-2001<br>CBP 2002-2015	
**Switzerland**	Before a reform in 2012, hospitals received per diem payments combined with fixed	Mixed 1994-2004	x		

	budgets. In general, Swiss hospital funding is subject to large regional variations. Nationwide Swiss-DRG started in 2012; before this there were regional DRG implementations starting in 2003 (mainly related to budget only). [57, 58]	CBP 2012-2015			
**United Kingdom (England)**	Before 2003, hospitals were mainly paid by annual fixed budgets. A DRG system was introduced in 2003 and became operational in 2005. [27, 59]	FB 1994-2004<br>CBP 2005-2015			

TABLE SI 1. Hospital payment scheme classification

CG = Control Group

Data collection process

We use unbalanced country-level panel data from OECD sources, complemented by data from Eurostat [60, 61] and for economic indicators from the World Bank [62].

Our main outcomes of interest were related to hospital activity and efficiency, which we operationalized as hospital discharges and average length of hospital stay, respectively. Secondary outcome variables were related to hospital resources and expenditure, as well as population health status. We used inpatient expenditure as a measure of hospital expenditure, but the data availability was poor. We used the number of physicians and nurses employed by hospitals to operationalize hospital resources. However, the data quality and availability were rather poor as well. As indicators for overall population health status, we used standardized all-cause death rates, life expectancy and potential years of life lost.

For some of our variables, there were different closely related data definitions. These differed, for example, with respect to the types of considered hospital (curative vs. curative and rehabilitative) or the unit of measurement (full-time equivalents vs. head counts). We chose a main definition for each variable. When this definition was not available for a country or when it covered only a short period, we used closely related definitions with richer data. For example, instead of curative care discharges, we used inpatient (curative and rehabilitative) discharges for some countries. See

Variable	Data definition
**Hospital discharges per 100,000 inhabitants**	Main:<br>• Curative (acute) care discharges *(HEALTH_PROC)*<br>Additional:<br>• Inpatient care discharges (All hospitals) (CZE, DNK, LUX, POL) *(HEALTH_PROC)*<br>• All causes discharges - Hospital discharges by diagnostic categories (AUS, NLD) *(HEALTH_PROC)*<br>• Hospital discharges for curative care (Eurostat)
**Average length of stay**	Main:<br>• Curative care average length of stay *(HEALTH_PROC)*<br>Additional:<br>• Inpatient care average length of stay (All hospitals) - (AUS, CZE, DNK, ISL, NOR) *(HEALTH_PROC)*<br>• Average length of stay for inpatient care (Eurostat)
**Share of population aged 65 years or older**	Main:<br>• Population 65 years old or older as percent of total population *(HEALTH_DEMR)*<br>Additional:<br>• Proportion of population aged 65 years or older (Eurostat)

**Gross domestic product (GDP) per capita**	Main:<br>• GDP per capita, PPP (constant 2011 international $) (World Bank)<br>Additional:<br>• None
**Inpatient expenditure**	Main:<br>• Inpatient curative and rehabilitative care expenditure (HC11HC21) for all healthcare providers (HPTOT) per capita, constant prices, constant PPPs, OECD base year *(SHA)*<br>Additional:<br>• Inpatient curative and rehabilitative care expenditure (HC11_21) for all healthcare providers (TOTAL) PPS per inhabitant (Eurostat)
**Outpatient Expenditure**	Main:<br>• Outpatient curative and rehabilitative care expenditure (HC13HC23) for all providers (HPTOT) per capita, constant prices, constant PPPs, OECD base year *(SHA)*<br>Additional:<br>• Outpatient curative and rehabilitative care expenditure (HC13_23) for all providers (TOTAL) PPPs per inhabitant (Eurostat)
**Hospital expenditure**	Main:<br>• All healthcare expenditure (HCTOT) for all hospitals (HP1) per capita, constant prices, constant PPPs, OECD base year *(SHA)*<br>Additional:<br>• All healthcare expenditure (TOT_HC) for all hospitals (HP1) PPS per inhabitant (Eurostat)
**Healthcare expenditure**	Main:<br>• All healthcare expenditure (HCTOT) for all providers (HPTOT) per capita, constant prices, constant PPPs, OECD base year *(SHA)*<br>Additional:<br>• All healthcare expenditure (TOT_HC) for all providers (TOTAL) PPPs per inhabitant (Eurostat)
**Hospital physicians and nursing professionals (including associates) per 1000 inhabitants**	Main:<br>• Total hospital employment in full time equivalents *(HEALTH_REAC)*<br>Additional:<br>• Total hospital employment in head counts (CAN, DNK, ESP, GRC, ITA, LVA, NLD, POL, POR, SVN) *(HEALTH_REAC)*<br>• Health personnel employed in hospital in head counts (Eurostat)
**Hospital beds per 1000 inhabitants**	Main:<br>• Curative (acute) care beds *(HEALTH_REAC)*<br>Additional:<br>• Total hospital beds (AUS, GBR, GRC, POR)<br>• Available beds in hospitals (Eurostat)
**Private hospital beds per 1000 inhabitants**	Main:<br>• Beds in private for-profit hospitals *(HEALTH_REAC)*<br>Additional:<br>• Hospital beds by hospital ownership – private for-profit (Eurostat)

**Bed occupancy rate and idle bed capacity**	Main:<br>• Curative (acute) care occupancy rate *(HEALTH_PROC)*<br>Additional:<br>• Curative care bed occupancy rate (Eurostat)<br><br>*Idle bed capacity = [1- bed occupancy rate] * hospital beds*
**Life expectancy at birth / at 65**	Main:<br>• Life expectancy total population at birth / at 65 (*HEALTH_STAT*)<br>Additional:<br>• Life expectancy in the age class "less than one year" / 65 (Eurostat)
**Standardized death rates per 100,000 inhabitants**	Main:<br>• All causes of death per 100,000 population (standardized rates) (*HEALTH_STAT*)<br>Additional:<br>• Standardized death rate for all ages (Eurostat)
**Potential years of life lost per 100,000 inhabitants**	Main:<br>• Potential years of life lost - years lost per 100,000 population aged 0 to 75 (*HEALTH_STAT*)<br>o All causes<br>Additional:<br>• None
**Unemployment rate**	Main:<br>• Unemployment, total (% of total labor force) (modeled ILO estimate) (World Bank)<br>Additional:<br>• None
**Non-hospital ambulatory care consultations**	Main:<br>• Doctor consultations in all settings per capita [all settings do not include inpatient] *(HEALTH_PROC)*<br>Additional:<br>• Consultation of a medical doctor (in private practice or as outpatient) per inhabitant (Eurostat)

TABLE SI 2 for an overview of the definitions and the countries for which additional data definitions were used. Data for Cyprus were added from Eurostat.

Due to our data collection process, data definitions can differ slightly from country to country but do not differ over time within any given country. This approach is suitable for two reasons. First, countries often deviate from the main definition when reporting aggregate data. This is why OECD data are accompanied by extensive data definition sheets with country-specific inclusion and exclusion criteria. The additional variation induced by using closely related data definitions therefore seemed acceptable. Second, since the focus of our analyses was differences over time, varying data definitions – across countries but not over time – should not bias our results.

Variable	Data definition
**Hospital discharges per 100,000 inhabitants**	Main:<br>• Curative (acute) care discharges *(HEALTH_PROC)*<br>Additional:<br>• Inpatient care discharges (All hospitals) (CZE, DNK, LUX, POL) *(HEALTH_PROC)*<br>• All causes discharges - Hospital discharges by diagnostic categories (AUS, NLD) *(HEALTH_PROC)*<br>• Hospital discharges for curative care (Eurostat)
**Average length of stay**	Main:<br>• Curative care average length of stay *(HEALTH_PROC)*<br>Additional:<br>• Inpatient care average length of stay (All hospitals) - (AUS, CZE, DNK, ISL, NOR) *(HEALTH_PROC)*<br>• Average length of stay for inpatient care (Eurostat)
**Share of population aged 65 years or older**	Main:<br>• Population 65 years old or older as percent of total population *(HEALTH_DEMR)*<br>Additional:<br>• Proportion of population aged 65 years or older (Eurostat)
**Gross domestic product (GDP) per capita**	Main:<br>• GDP per capita, PPP (constant 2011 international $) (World Bank)<br>Additional:<br>• None
**Inpatient expenditure**	Main:<br>• Inpatient curative and rehabilitative care expenditure (HC11HC21) for all healthcare providers (HPTOT) per capita, constant prices, constant PPPs, OECD base year *(SHA)*<br>Additional:<br>• Inpatient curative and rehabilitative care expenditure (HC11_21) for all healthcare providers (TOTAL) PPS per inhabitant (Eurostat)
**Outpatient Expenditure**	Main:<br>• Outpatient curative and rehabilitative care expenditure (HC13HC23) for all providers (HPTOT) per capita, constant prices, constant PPPs, OECD base year *(SHA)*<br>Additional:<br>• Outpatient curative and rehabilitative care expenditure (HC13_23) for all providers (TOTAL) PPPs per inhabitant (Eurostat)
**Hospital expenditure**	Main:<br>• All healthcare expenditure (HCTOT) for all hospitals (HP1) per capita, constant prices, constant PPPs, OECD base year *(SHA)*<br>Additional:<br>• All healthcare expenditure (TOT_HC) for all hospitals (HP1) PPS per inhabitant (Eurostat)

**Healthcare expenditure**	Main:<br>• All healthcare expenditure (HCTOT) for all providers (HPTOT) per capita, constant prices, constant PPPs, OECD base year *(SHA)*<br>Additional:<br>• All healthcare expenditure (TOT_HC) for all providers (TOTAL) PPPs per inhabitant (Eurostat)
**Hospital physicians and nursing professionals (including associates) per 1000 inhabitants**	Main:<br>• Total hospital employment in full time equivalents *(HEALTH_REAC)*<br>Additional:<br>• Total hospital employment in head counts (CAN, DNK, ESP, GRC, ITA, LVA, NLD, POL, POR, SVN) *(HEALTH_REAC)*<br>• Health personnel employed in hospital in head counts (Eurostat)
**Hospital beds per 1000 inhabitants**	Main:<br>• Curative (acute) care beds *(HEALTH_REAC)*<br>Additional:<br>• Total hospital beds (AUS, GBR, GRC, POR)<br>• Available beds in hospitals (Eurostat)
**Private hospital beds per 1000 inhabitants**	Main:<br>• Beds in private for-profit hospitals *(HEALTH_REAC)*<br>Additional:<br>• Hospital beds by hospital ownership – private for-profit (Eurostat)
**Bed occupancy rate and idle bed capacity**	Main:<br>• Curative (acute) care occupancy rate *(HEALTH_PROC)*<br>Additional:<br>• Curative care bed occupancy rate (Eurostat)<br><br>*Idle bed capacity = [1- bed occupancy rate] * hospital beds*
**Life expectancy at birth / at 65**	Main:<br>• Life expectancy total population at birth / at 65 (*HEALTH_STAT*)<br>Additional:<br>• Life expectancy in the age class "less than one year" / 65 (Eurostat)
**Standardized death rates per 100,000 inhabitants**	Main:<br>• All causes of death per 100,000 population (standardized rates) (*HEALTH_STAT*)<br>Additional:<br>• Standardized death rate for all ages (Eurostat)
**Potential years of life lost per 100,000 inhabitants**	Main:<br>• Potential years of life lost - years lost per 100,000 population aged 0 to 75 (*HEALTH_STAT*)<br>○ All causes<br>Additional:<br>• None

**Unemployment rate**	Main:<br>• Unemployment, total (% of total labor force) (modeled ILO estimate) (World Bank)<br>Additional:<br>• None
**Non-hospital ambulatory care consultations**	Main:<br>• Doctor consultations in all settings per capita [all settings do not include inpatient] *(HEALTH_PROC)*<br>Additional:<br>• Consultation of a medical doctor (in private practice or as outpatient) per inhabitant (Eurostat)

TABLE SI 2: Data definitions

PPP = purchasing power parity

Missing data were, where justifiable, imputed using time series imputation [63]. We considered a period consisting of a maximum of three missing years to be justifiable for imputation. The following figures illustrate data availability and imputed data points.

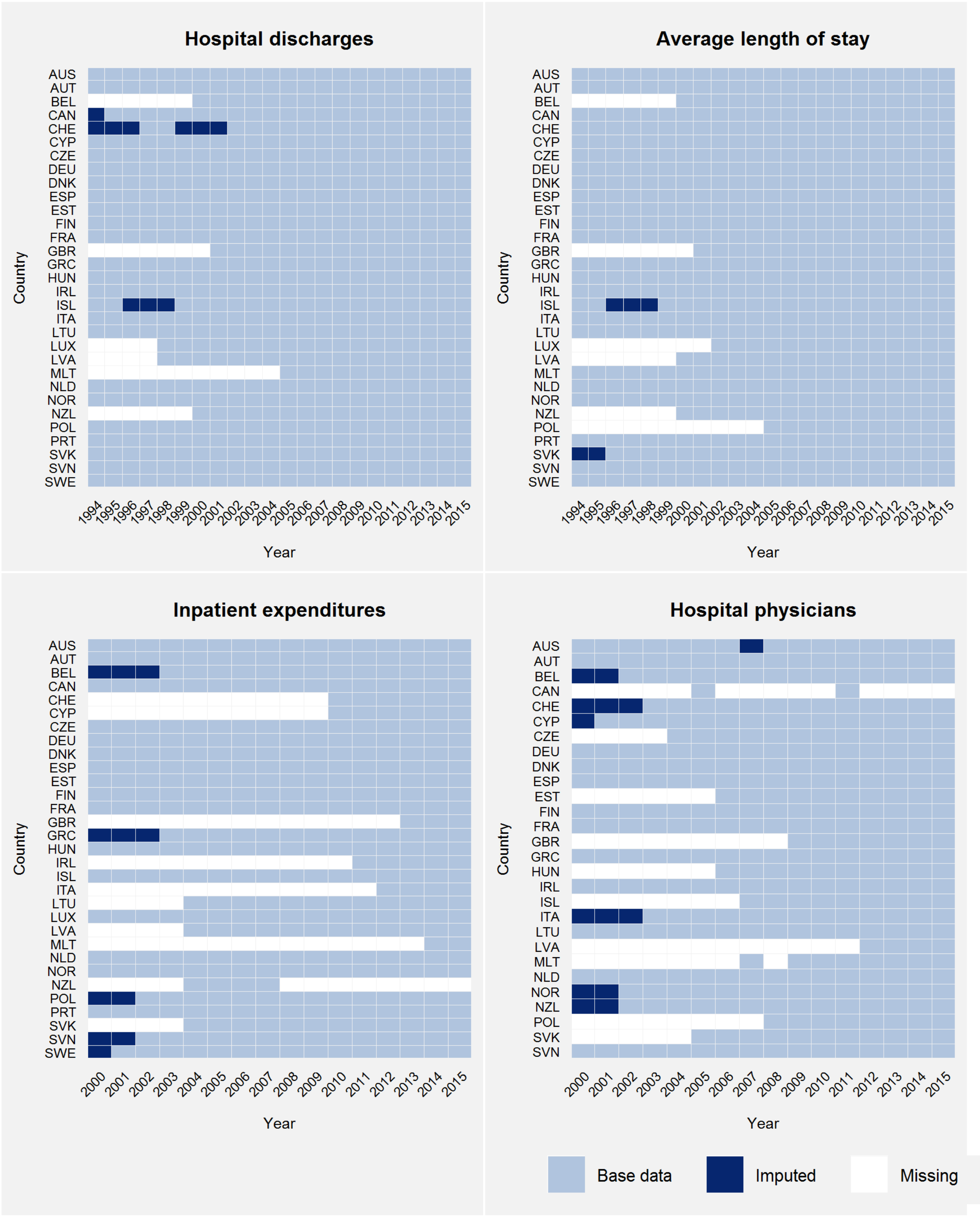

FIGURE SI 1. Data availability for outcomes – Part 1

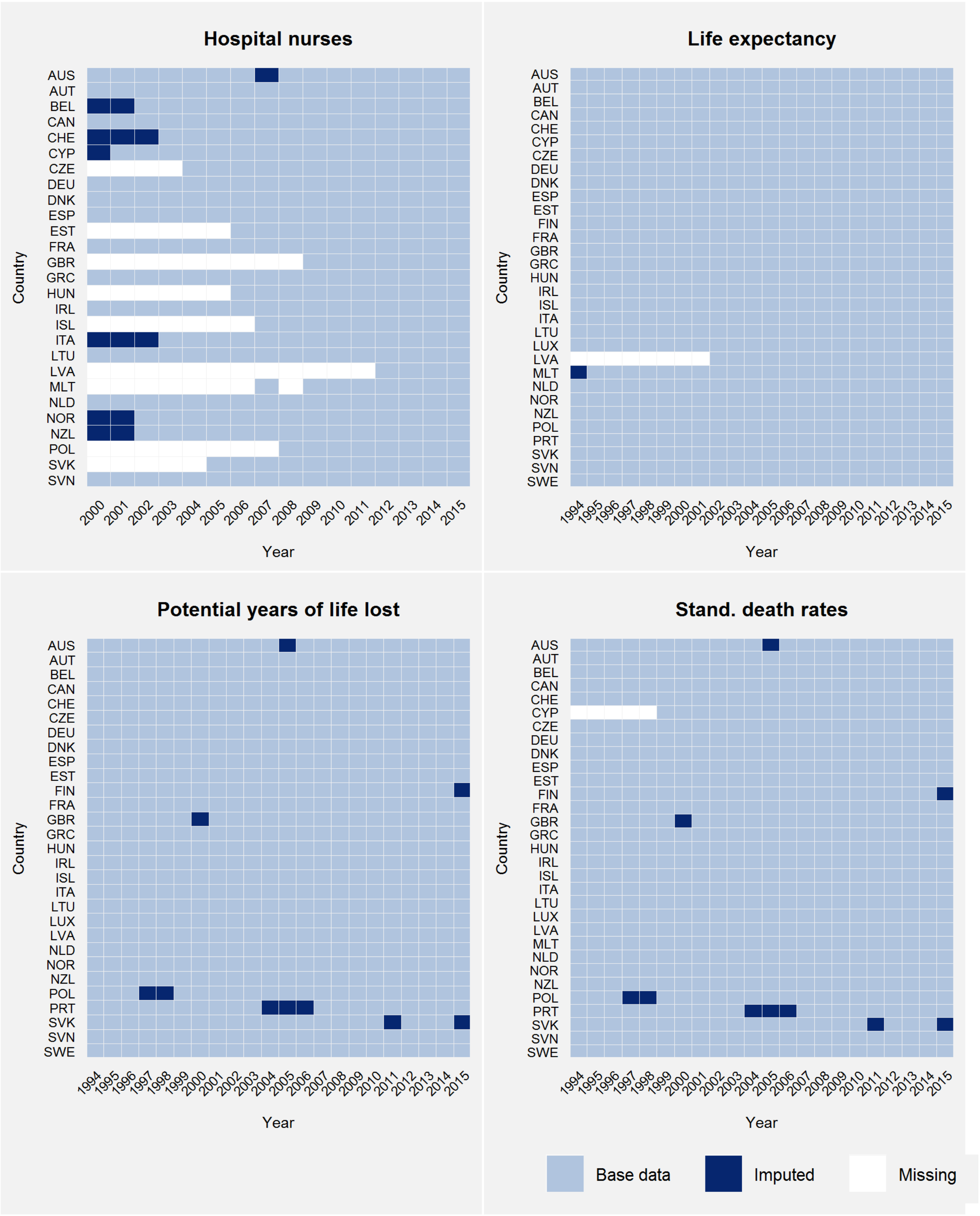

FIGURE SI 2. Data availability for outcomes – Part 2

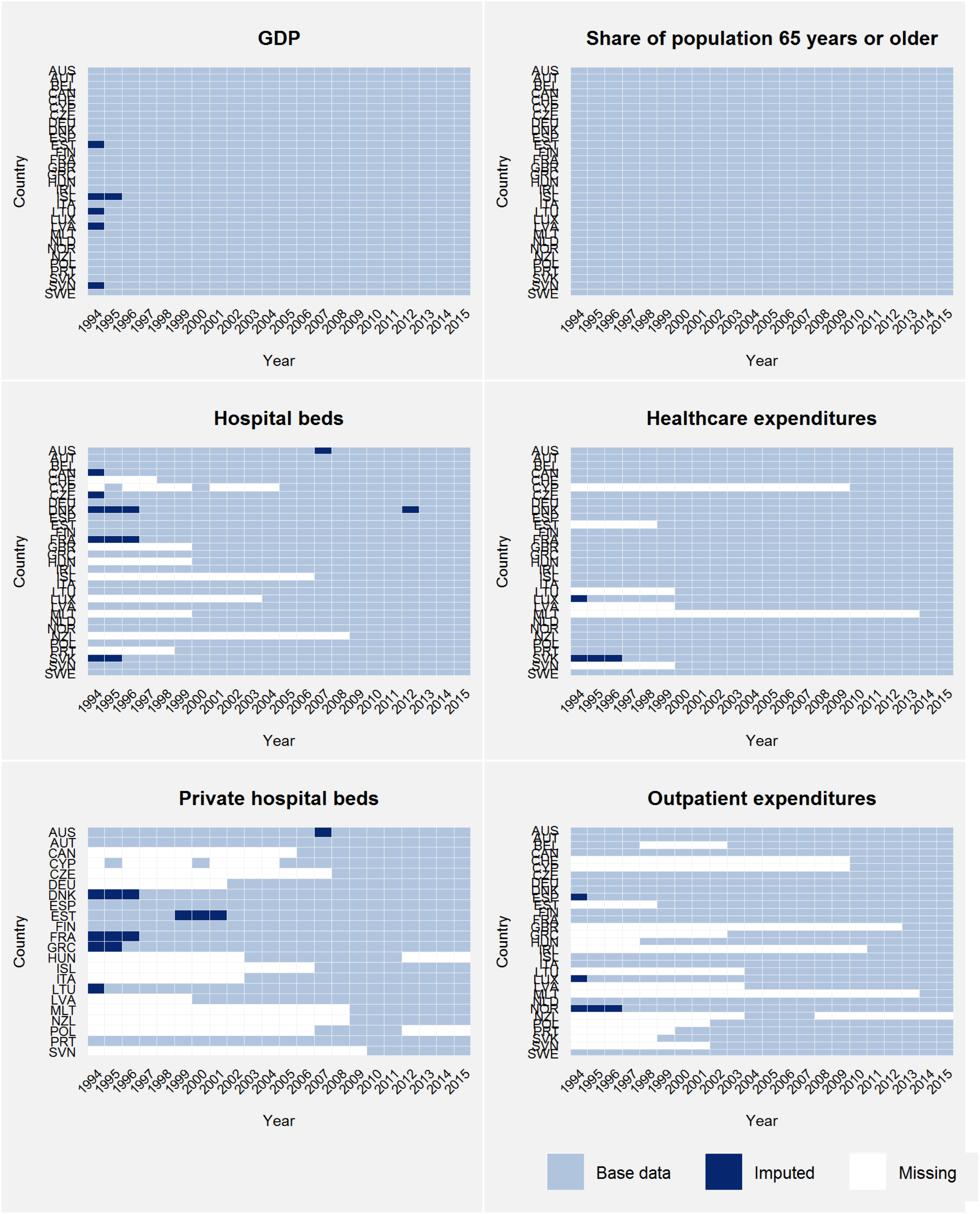

FIGURE SI 3. Data availability for control variables

Supporting Information References

1. Moreno-Serra, R., Wagstaff, A.: System-wide impacts of hospital payment reforms: evidence from Central and Eastern Europe and Central Asia. J Health Econ (2010). https://doi.org/10.1016/j.jhealeco.2010.05.007
2. Wubulihasimu, P., Brouwer, W., van Baal, P.: The Impact of Hospital Payment Schemes on Healthcare and Mortality: Evidence from Hospital Payment Reforms in OECD Countries. Health Econ (2016). https://doi.org/10.1002/hec.3205
3. Kroneman, M., Nagy, J.: Introducing DRG-based financing in Hungary: a study into the relationship between supply of hospital beds and use of these beds under changing institutional circumstances. Health Policy (2001). https://doi.org/10.1016/S0168-8510(00)00118-4
4. OECD: Health Systems Characteristics Survey. OECD. https://qdd.oecd.org/Home/ (2016)
5. Paris, V., Devaux, M., Wei, L.: Health Systems Institutional Characteristics: A Survey of 29 OECD Countries. OECD Health Working Papers No. 50 (2010)
6. WHO: The Health Systems in Transition (HiT) Series. European Observatory on Health System and Policies. https://eurohealthobservatory.who.int/publications/health-systems-reviews (2021)
7. Parliamentary Library: Budget Review 2016-17. Research paper Series, 2015-16. https://www.aph.gov.au/About_Parliament/Parliamentary_Departments/Parliamentary_Library/pubs/rp/BudgetReview201617/Hospital (2016)
8. Hilless, M., Healy, J.: Australia: Health system review. Health Syst Transit **13** (2001)
9. Theurl, E.: Reform of hospital financing in Austria: successes, failures, and the way forward. Eur J Health Econ (2015). https://doi.org/10.1007/s10198-014-0641-1
10. Stephani, V., Quentin, W., van den Heede, K., van de Voorde, C., Geissler, A.: Payment methods for hospital stays with a large variability in the care process. Health Services Research (HSR) Brussels: Belgian Health Care Knowledge Centre (KCE) 302 (2018)
11. Gerkens, S., Merkur, S.: Belgium: Health system review. Health Syst Transit **12** (2010)
12. Sutherland, J., Repin, N.: Current Hospital Funding in Canada. Policy Brief. Vancouver: UBC Centre for Health Services and Policy Research. www.healthcarefunding.ca (2014)
13. Sutherland, J., Liu, G., Crump, R.T., Law, M.: Paying for volume: British Columbia's experiment with funding hospitals based on activity. Health Policy (2016). https://doi.org/10.1016/j.healthpol.2016.09.010
14. Marchildon, G.P., Allin, S., Merkur, S.: Canada: Health system review. Health Syst Transit **22** (2020)
15. Theodorou, M., Charalambous, C., Petrou, C., Cylus, J.: Cyprus: health system review. Health Syst Transit **14** (2012)

16. Kotherová, Z., Caithamlová, M., Nemec, J., Dolejšová, K.: The Use of Diagnosis-Related Group-Based Reimbursement in the Czech Hospital Care System. Int J Environ Res Public Health (2021). https://doi.org/10.3390/ijerph18105463
17. Bryndová, L., Pavloková, K., Roubal, T., Rokosová, M., Gaskins, M., van Ginneken, E.: Czech Republic: health system review. Health Syst Transit **11** (2009)
18. Street, A., Vitikainen, K., Bjorvatn, A. and Hvenegaard, A.: Introducing activity-based financing: a review of experience in Australia, Denmark, Norway and Sweden. CHE Research Paper (2007)
19. Christiansen, T., Vrangbæk, K.: Hospital centralization and performance in Denmark-Ten years on. Health Policy (2018). https://doi.org/10.1016/j.healthpol.2017.12.009
20. Estonian Health Insurance Fund: Overview of Estonian experiences with DRG system. Department of Health Economics (2009)
21. Bredenkamp, C., Bales, S., Kahur, K. (eds.): Transition to diagnosis-related group (DRG) payments for health. Lessons from case studies. International development in focus. World Bank Group, Washington, DC, USA (2020)
22. Mathauer, I., Wittenbecher, F.: Hospital payment systems based on diagnosis-related groups: experiences in low- and middle-income countries. Bulletin of the World Health Organization (2013). https://doi.org/10.2471/BLT.12.115931
23. Lai, T., Habicht, T., Kahur, K., Reinap, M., Kiivet, R., van Ginneken, E.: Estonia: health system review. Health Syst Transit **15** (2013)
24. Stig, K., Paulsson Lütz, I.: Financing of Health Care in the Nordic Countries (2013)
25. Mikkola, H.: Hospital Pricing Reform in the Public Health Care System: An Empirical Case Study from Finland. International Journal of Health Care Finance and Economics **3** (2003)
26. Keskimäki, I., Tynkkynen, L.-K., Reissell, E., Koivusalo, M., Syrjä, V., Vuorenkoski, L., Rechel, B., Karanikolos, M.: Finland: health system review. Health Syst Transit **21** (2019)
27. van de Voorde, C., Gerkens, S., van den Heede, K., Swartenbroekx, N.: A comparative analysis of hospital care payments in five countries. KCE Reports, 207. Belgian Health Care Knowledge Centre (KCE), Brussels (2013)
28. Chevreul, K., Berg Brigham, K., Durand-Zaleski, I., Hernández-Quevedo, C.: France: health system review. Health Syst Transit **17** (2015)
29. Blümel, M., Spranger, A., Achstetter, K., Maresso, A., Busse, R.: Germany: Health system review. Health Syst Transit **22** (2020)
30. Polyzos, N., Karanikas, H., Thireos, E., Kastanioti, C., Kontodimopoulos, N.: Reforming reimbursement of public hospitals in Greece during the economic crisis: Implementation of a DRG system. Health Policy (2013). https://doi.org/10.1016/j.healthpol.2012.09.011
31. Economou, C., Kaitelidou, D., Karanikolos, M., Maresso, A.: Greece: health system review. Health Syst Transit **19** (2017)

32. Endrei, D., Zemplényi, A., Molics, B., Agoston, I., Boncz, I.: The effect of performance-volume limit on the DRG based acute care hospital financing in Hungary. Health Policy (2014). https://doi.org/10.1016/j.healthpol.2013.12.005
33. Gaál, P., Szigeti, S., Csere, M., Gaskins, M., Panteli, D.: Hungary: health system review. Health Syst Transit **13** (2011)
34. McElroy, B., Murphy, A.: An economic analysis of money follows the patient. Irish journal of medical science (2014). https://doi.org/10.1007/s11845-013-1050-7
35. Health Service Executive: Activity Based Funding (ABF) Programme Implementation Plan 2021 to 2023. https://www.hse.ie/eng/services/publications/activity-based-funding-abf-programme-implementation-plan-2021-2023.pdf (2021)
36. McDaid, D., Wiley, M., Maresso, A., Mossialos, E.: Ireland: health system review. Health Syst Transit **11** (2009)
37. Anessi-Pessina, E., Nieddu, L., Rizzo, M.G.: Does DRG funding encourage hospital specialization? Evidence from the Italian National Health Service. Int J Health Plann Manage (2019). https://doi.org/10.1002/hpm.2715
38. Cantù, E., Carbone, C., Anessi-Pessina, E.: 21. Do Italian regions effectively use DRG funding to steer provider behaviour? Policy, performance and management in governance and intergovernmental relations: transatlantic perspectives, 341 (2011)
39. Ferré, F., Belvis, A.G. de, Valerio, L., Longhi, S., Lazzari, A., Fattore, G., Ricciardi, W., Maresso, A.: Italy: health system review. Health Syst Transit **16** (2014)
40. Dubas-Jakóbczyk, K., Albreht, T., Behmane, D., Bryndova, L., Dimova, A., Džakula, A., Habicht, T., Murauskiene, L., Scîntee, S.G., Smatana, M., Velkey, Z., Quentin, W.: Hospital reforms in 11 Central and Eastern European countries between 2008 and 2019: a comparative analysis. Health Policy (2020). https://doi.org/10.1016/j.healthpol.2020.02.003
41. Behmane, D., Dudele, A., Villerusa, A., Misins, J., Klavina, K., Mozgis, D., Scarpetti, G.: Latvia: health system review. Health Syst Transit **21** (2019)
42. Murauskiene, L., Janoniene, R., Veniute, M., van Ginneken, E., Karanikolos, M.: Lithuania: health system review. Health Syst Transit **15** (2013)
43. Berthet, F., Calteux, A., Wolter, M., Weber, L., van Ginneken, E., Spranger, A.: Luxembourg: HiT in brief. Health Syst Transit (2015)
44. Azzopardi-Muscat, N., Buttigieg, S., Calleja, N., Merkur, S.: Malta: Health system review. Health Syst Transit **19** (2017)
45. Cumming, J., McDonald, J., Barr, C., Martin, G., Gerring, Z., Daubé, J.: New Zealand health system review. Health Syst Transit **4** (2014)
46. Schut, F.T., van de Ven, W.P.M.M.: Rationing and competition in the Dutch health-care system. Health Econ (2005). https://doi.org/10.1002/hec.1036
47. Krabbe-Alkemade, Y.J.F.M., Groot, T.L.C.M., Lindeboom, M.: Competition in the Dutch hospital sector: an analysis of health care volume and cost. Eur J Health Econ (2017). https://doi.org/10.1007/s10198-016-0762-9
48. Kroneman, M., Boerma, W.G., van den Berg, M., Groenewegen, P., Jong, J. de, van Ginneken, E.: Netherlands: health system review. Health Syst Transit **18** (2016)

49. Sperre Saunes, I., Karanikolos, M., Sagan, A.: Norway: health system review. Health Syst Transit **22** (2020)
50. Czach, K., Klonowska, K., Swiderek, M., Wiktorza, K.: Poland: the Jednorodne Grupy Pacjentów-Polish experiences with DRGs. In: Busse, R., Geissler, A., Quentin, W., Wiley, M.M. (eds.) Diagnosis-related groups in Europe. Moving towards transparency, efficiency and quality in hospitals. European Observatory on Health Systems and Policies series, pp. 359–380. Open University Press, Maidenhead, England (2011)
51. Sowada, C., Sagan, A., Kowalska-Bobko, I., Badora-Musiał, K., Bochenek, T., Domagała, A., Dubas-Jakóbczyk, K., Kocot, E., Mrożek-Gąsiorowska, M., Sitko, S.J., Szetela, A.M., Szetela, P., Tambor, M., Więckowska, B., Zabdyr-Jamróz, M.: Poland: health system review. Health Syst Transit **21** (2019)
52. Simões, J., Augusto, G.F., Fronteira, I., Hernández-Quevedo, C.: Portugal: Health system review. Health Syst Transit **19**, 1–184 (2017)
53. Smatana, M., Pažitný, P., Kandilaki, D., Laktišová, M., Sedláková, D., Palušková, M., van Ginneken, E., Spranger, A.: Slovakia: health system review. Health Syst Transit **18** (2016)
54. Albreht, T., Polin, K., Brinovec, R.P., Kuhar, M., Poldrugovac, M., Rehberger, P.O., Rupel, V.P., Vracko, P.: Slovenia: health system review. Health Syst Transit **23** (2021)
55. Bernal-Delgado, E., Garcia-Armesto, S., Oliva, J., Sanchez Martinez, F.I., Repullo, J.R., Pena-Longobardo, L.M., Ridao-Lopez, M., Hernandez-Quevedo, C.: Spain: Health System Review. Health Syst Transit **20**, 1–179 (2018)
56. Anell, A., Glenngård, A.H., Merkur, S.: Sweden: Health system review. Health Syst Transit **14** (2012)
57. Busato, A., Below, G. von: The implementation of DRG-based hospital reimbursement in Switzerland: A population-based perspective. Health Res Policy Syst (2010). https://doi.org/10.1186/1478-4505-8-31
58. Pietro, C. de, Camenzind, P., Sturny, I., Crivelli, L., Edwards-Garavoglia, S., Spranger, A., Wittenbecher, F., Quentin, W.: Switzerland: health system review. Health Syst Transit **17** (2015)
59. Cylus, J., Richardson, E., Findley, L., Longley, M., O'Neill, C., Steel, D.: United Kingdom: health system review. Health Syst Transit **17** (2015)
60. OECD: Health statistics. https://doi.org/10.1787/data-00900-en (2021)
61. Eurostat: Health Database. https://ec.europa.eu/eurostat/web/health/data/database (2021)
62. World Bank: World Bank Open Data. https://data.worldbank.org/ (2021)
63. Moritz, S., Bartz-Beielstein, T.: imputeTS: Time Series Missing Value Imputation in R. The R Journal (2017). https://doi.org/10.32614/RJ-2017-009
